# Development and validation of the Interoceptive States Vocalisations (ISV) and Interoceptive States Point Light Displays (ISPLD) databases

**DOI:** 10.3758/s13428-024-02514-0

**Published:** 2025-03-31

**Authors:** Federica Biotti, Lily Sidnick, Anna L Hatton, Diar Abdlkarim, Alan Wing, Janet Treasure, Francesca Happé, Rebecca Brewer

**Affiliations:** 1 Queen Mary, University of London, London, UK; 2 Royal Holloway, University of London, Egham Hill, Egham, TW20 0EX UK; 3 The University of Queensland, Brisbane, Australia; 4 University of Birmingham, Birmingham, UK; 5 King’s College London, London, UK

**Keywords:** Interoception, Social perception, Vocalisations, Point light displays, Stimulus development

## Abstract

The ability to perceive others’ emotions and one’s own interoceptive states has been the subject of extensive research. Very little work, however, has investigated the ability to recognise others’ interoceptive states, such as whether an individual is feeling breathless, nauseated, or fatigued. This is likely owing to the dearth of stimuli available for use in research studies, despite the clear relevance of this ability to social interaction and effective caregiving. This paper describes the development and validation of two stimulus sets for use in research into the perception of others’ interoceptive states. The Interoceptive States Vocalisations (ISV) database and the Interoceptive States Point Light Displays (ISPLD) database include 191 vocalisation and 159 point light display stimuli. Both stimulus sets underwent two phases of validation, and all stimuli were scored in terms of their quality and recognisability, using five different measures. The ISV also includes control stimuli featuring non-interoceptive vocalisations. Some interoceptive states were consistently recognised better than others, but variability was observed within, as well as between, stimulus categories. Stimuli are freely available for use in research, and are presented alongside all stimulus quality scores, in order for researchers to select the most appropriate stimuli based on individual research questions.

**Supplementary information:**

The online version contains supplementary material available at 10.3758/s13428-024-02514-0.

## Introduction

Interoception refers to the ability to perceive, and tendency to attend to, internal signals from the body, such as cardiac, respiratory, and gastric signals (Craig, 2009). Interoception has received substantial research attention in recent decades, owing in part to the role it plays in multiple psychological processes, such as emotion processing (Pace-Schott et al., 2019), and learning and decision making (e.g. Damasio, 1994; Werner et al., 2009), as well as its notable relationship with mental health; indeed, atypical interoception has been observed across a wide range of conditions, such as autism, eating disorders, depression, and anxiety (Brewer et al., 2021; Khalsa et al., 2017). While research has focused on individual differences in the perception of one’s own interoceptive signals, very little empirical work has investigated the ability to detect and interpret interoceptive signals in others.

Interoception is closely associated with emotional experience. Both traditional and contemporary models of emotion assign a role for physiological signals in giving rise to emotions (James, 1894; Lange, 1885; Schachter & Singer, 1962), and participants report interoceptive sensations as being a key feature of emotions (Ferré et al., 2023) as well as confusing bodily sensations with emotions (Brewer et al., 2016). One’s ability to perceive one’s interoceptive signals appears to be closely associated with the intensity of one’s emotional experience (Parrinello et al., 2022), and the ability to recognise and empathise with others’ emotions (Chick et al., 2019; Georgiou et al., 2018; Terasawa et al., 2014). Further, there is substantial overlap in the neural substrates supporting interoception and emotion (Critchley & Garfinkel, 2017; Quadt et al., 2018), with the anatomy of interoceptive pathways thought to give rise to affective responses (Feldman et al., 2024). Within the emotional domain, several studies have investigated humans’ ability to recognise others’ emotions. The vast majority of work has used static image stimuli depicting emotional facial or body expressions (e.g. Ekman & Freisen, 1976; Langner et al., 2010; Lundqvist et al., 1998; Volkova et al., 2014; Wingenbach et al., 2016), but research on emotion recognition has also made use of vocalisations (e.g. Biotti & Cook, 2016; Cowen et al., 2019; Lima et al., 2013; Simon-Thomas et al., 2009), film clips (Goodkind et al., 2015; Lucey et al., 2010; Richter et al., 2011; Werner et al., 2007) and kinematic information, for example in facial or full body point light displays (e.g. Alaerts et al., 2011; Bidet-Ildei et al., 2020; Lorey et al., 2012; Mazzoni et al., 2022; Sowden et al., 2021). The use of a range of stimuli, spanning various modalities, has been invaluable within the field of emotion recognition, as it has allowed investigation into the similarities and differences in emotion processing across modalities, as well as the mechanisms underlying and benefits of integration of congruous multimodal cues to others’ emotion (Schirmer & Adolphs, 2017). Previous studies have sought to explore the way in which emotional information from one modality biases interpretation of emotion cues in another modality, the time course and neural basis of cross-modal integration, developmental trajectories of multimodal cue processing, and individual differences in processing of emotion cues across modalities, in both typical and clinical populations (e.g. Brewer, Biotti, Bird, & Cook, 2017; Campanella & Belin, 2007; Kucharska-Pietura et al., 2004; Ross et al., 2012; Van den Stock et al., 2007; Zhang et al., 2022).

Despite the multitude of stimuli available to investigate social perception within the emotional domain, there is a notable lack of stimuli depicting interoceptive states, such as hunger, fatigue, nausea, and breathlessness. The ability to recognise these states in others is important for social interactions and relationships, as well as for providing care to others, both within social and family settings, and in health and medical contexts. If one can identify signals of states such as nausea, hunger, pain, fatigue, and cold in others, one can respond with appropriate care. Recent studies have assessed the ability to detect others’ pain (e.g. Brewer et al., 2015), illness (Axelsson et al., 2018), or heart rate (Galvez-Pol et al., 2022), and others have investigated recognition of babies’ cries, in terms of inferences of pain or sickness (LaGasse et al., 2005; Schuetze & Zeskind, 2001; Zeskind & Lester, 1978), yet only one set of controlled stimuli depicting a range of interoceptive states exists. We recently published the ISSI database of static images depicting actors expressing breathlessness, cold, fatigue, hotness, hunger, itch, nausea, pain, and satiety (Biotti et al., 2022), enabling researchers to investigate the ability to process others’ interoceptive states. There is a need, however, to investigate this ability across multiple modalities. This is particularly true for populations who experience difficulties when interpreting social information from static images of facial and bodily expressions. Autistic individuals, for example, may exhibit atypical processing of interoceptive cues from images of others, owing to differing patterns of attention to faces and bodies (Chita-Tegmark, 2016) or holistic processing (Naumann et al., 2018). Alternative sources of information, such as vocalisation, or kinematic information from point light displays (dynamic arrays of light points associated with an individual’s joint locations), may reduce the impact of atypical visual attention to faces in autism when recognising others’ interoceptive states (although individual differences in holistic processing may still contribute to point light display processing). It is also likely that developmental trajectories differ for recognition of interoceptive cues across modalities, as is the case for emotion processing (e.g. Grossmann, 2010). The existence of multiple stimulus sets, with different perceptual properties, will therefore allow researchers to select appropriate stimuli based on the research question of interest. Further, multiple stimulus sets across different modalities will allow for investigation of questions such as the extent to which different interoceptive cues are weighted, whether cues from different modalities compete with or facilitate recognition of each other, and whether these effects vary across individuals or groups.

The current paper presents the development and validation of two new stimulus sets, namely the Interoceptive States Vocalisations (ISV) database and the Interoceptive States Point Light Displays (ISPLD) database. The ISV database presents 191 auditory stimuli (1–6 seconds) produced by 12 actors expressing seven interoceptive states. The ISV database also includes 108 matched control stimuli, produced by the same actors performing five different control vocalisations. The ISPLD database presents 159 (5-second) video stimuli depicting 10 actors expressing nine interoceptive states through full body motion. Personally identifying information is largely removed, as the stimuli consist only of 16 moving points, corresponding to key body locations. Stimuli for both stimulus sets were validated in two stages (stage 1 utilised free labelling; stage 2 utilised a rating scale). Recognition data are presented for the individual stimuli, and comparisons across stimulus types and categories are reported.

## Interoceptive states vocalisations database

### Vocal stimulus development

#### Actors

Twelve trained adult actors (six male, six female) were recruited via online and campus advertisements. The actors were either professionals who had completed acting training or drama students at Royal Holloway, University of London (RHUL). Actors gave informed consent for their vocalisations to be recorded and made publicly available for use in research studies, shared with the scientific community, and presented at public talks and conferences. A financial remuneration was given to all actors for their time.

#### Procedure

Prior to attending the recording session, actors were provided with a list of interoceptive state and control vocalisations that they would be asked to produce, and permitted time to practice the vocalisations in advance. Vocalisations were then recorded in a soundproofed recording studio using a microphone connected to Audacity, an audio editing and recording software package.

Actors first produced ten control vocalisations (kissing, chewing, humming, tongue clicking, whistling, blowing, the phonetic sound *ɑ:* as in ‘car’, the phonetic sound *ɔ:* as in ‘law’, the phonetic sound *i:* as in ‘need’, and the phonetic sound *u:* as in ‘boot’), followed by vocalisations expressing ten interoceptive states (cold, fatigue, nausea, pain, breathlessness, hunger, thirst, hotness, satiety, and itch). Interoceptive states were selected from a list of states that have either been described as interoceptive (Khalsa & Lapidus, 2016; Khalsa et al., 2017) or are associated with activation in the insula (e.g., Critchley & Harrison, 2013; Langer et al., 2010; Mazzone et al., 2007), an area consistently associated with the processing of interoceptive signals. The states utilised in the final stimulus set were those that it was deemed likely that actors would be able to express using recognisable vocal cues. Control vocalisations were selected in order to not contain linguistic information with semantic content (i.e. no words), and not be associated with interoceptive signals. Before recording each stimulus type (e.g. cold, chewing), actors practised the vocalisation again in the recording booth. Actors then produced five exemplars of each stimulus type, allowing for a 5-second pause between each attempt. A short break was given following each stimulus type, to allow actors to drink water and rest their voice. Four of the 12 actors produced only interoceptive state stimuli, so control stimuli are only available from eight actors.

#### Stimulus editing and selection

Vocal recordings were edited in Audacity. For each stimulus type, the five exemplar vocalisations from each actor were saved as separate audio files. Each audio file was edited to remove background noise, audio artefacts (e.g., reverberation, clipping), and any unwanted sound (e.g., laughter, and verbal expressions such as ‘ouch’ when expressing pain). The resulting vocalisations were assessed by the researchers, and a vocal communication expert, for quality and systematic differences across stimulus categories.

Three internal states (i.e., thirst, itch, and hunger) were removed from the stimulus set due to reports from all actors that vocal expressions of these states were very difficult to produce. The four vowel sounds (ɑ:, ɔ:, i:, and u:) in the control vocalisations set were also removed following identification of systematic differences in the vocal production of the stimuli from the two stimulus categories; the internal states tended to show bursts of inhalation/exhalation patterns, whilst vowel sounds were often characterised by single sustained exhalations. The remaining control vocalisations therefore all corresponded to actions. Finally, the control vocalisation ‘blowing’ was also removed from the set due to these vocalisations presenting many audio artefacts that could not be removed artificially without degrading the quality of the intended vocalisation. To reduce the number of stimuli, only the three highest quality (in terms of auditory properties) exemplars of each stimulus type for each actor were selected for validation. Where three high-quality exemplars were not obtained, only two exemplars were retained. This yielded a total of 363 stimuli.

### Vocal stimulus validation

#### Phase 1: Free-labelling task

Fifteen participants (one male, 14 female) aged 18–27 years (*M* = 19.39, *SD* = 2.12) were recruited through the RHUL SONA system (participant recruitment system) to participate in a free-labelling task. All participants were students at RHUL and received course credits for participating. To participate in this validation study, participants were required to have no hearing impairment and be fluent in English. Participants were not informed of the purpose of the study prior to taking part, to avoid discussion of interoceptive states influencing their responses, but were debriefed following participation. Participants were required to listen to and describe each vocal stimulus in a free-labelling procedure. Participants received both written and verbal descriptions of the task procedure. Verbal instructions were standardised across participants and reported verbatim by the experimenter (FB), as follows:

*You will hear a series of vocalisations one by one. For each one, you need to provide a brief verbal description of what you think the vocalisation represents. There will be many stimuli, so it’s very important that you keep your answers as brief as possible. Ideally, you will use a single word or a short phrase. For example, if you hear a sneeze, you can simply answer ‘sneezing’. If you think that the vocalisation represents more than one thing, you can give multiple answers, but please try to keep the description of each brief. If I need more details, I will ask for them. There are not right or wrong answers, so I will not provide any feedback during or after the session. I will simply record your answers and occasionally intervene if I think something is not clear or if I need more details.*

Participants were given the opportunity to ask any questions about the procedure before commencing the free-labelling task. Each of the 363 stimuli was presented to participants through headphones, and the experimenter sat behind the participants and typed their responses verbatim. Standard phrases were used whenever additional information was required. If the answer needed further details, the experimenter would say ‘*Can you tell me more about that?’*. If the answer was unclear or ambiguous, the experimenter would say ‘*Can you be more specific?’* or ‘*Can you tell me what you mean by that?*’. Finally, if answers were too long, participants were reminded to ‘*Try to use single words or short phrases’*. The task was completed in 20–30 minutes per participant.

#### Phase 1: Free-labelling results

Participants’ free-labelling responses were coded by two independent researchers (SA, RQ). Responses were coded as ‘1’ if the participant used the intended state or control action label, or a semantically similar label, including associated behaviours, to describe the stimulus. For example, correct labels for the state ‘cold’ included descriptors such as ‘cold’, ‘feeling chilly’, and ‘shivering’. Labels that did not describe the intended stimulus type were coded as ‘0’. Where the two independent researchers disagreed, a third researcher (FB) was consulted until an agreement was reached. Inter-rater agreement between the two raters was high (*k* = .837).

A recognisability index (RI) (percentage of participants providing a correct label) was calculated for each stimulus (Appendix Table 5). Mean RIs for interoceptive state and control action stimuli were 47% and 70%, respectively. Of the internal state stimuli, RIs were highest for cold (*M = *67%, *SD* = 24%, range 13–100%) and nausea (*M = *66%, *SD* = 23%, range 13–100%) , whilst RIs were lowest for hotness (*M* = 1.5%, *SD* = 5%, range 0–27%) and satiety (*M* = 9%, *SD* = 13%, range 0–67%). Ranges indicate the individual stimuli with the lowest and highest RIs in each stimulus category. For the control action vocalizations, whistling (*M* = 87%, *SD* = 9%, range 60–93%) and kissing (*M* = 83%, *SD* = 17%, range 47–100%) were the best recognised, whilst humming (*M* = 57%, *SD* = 15%, range 13–80%) was the least well recognised. See Appendix Table 5 for RI for each stimulus.

As in the validation of the ISSI stimulus set (Biotti et al., 2022), stimuli were categorised according to RI as follows: very poor (RI scores 0.0–0.2), poor (RI scores 0.21–0.4), satisfactory (RI scores 0.41–0.6), good (RI scores 0.61–0.8), and very good (RI scores 0.81–1). All stimuli categorised as very good, good, and satisfactory were retained in the final database and rated in the second validation stage. In order to retain a minimum of two exemplars per actor for each stimulus type, where fewer than two stimuli within a stimulus type for a given actor were categorised as very good, good, or satisfactory, the two stimuli with the highest RIs were retained. This is consistent with the validation procedure used by Biotti et al. (2022). Twenty-five percent of the retained stimuli were categorised as poor or very poor, but it is worth noting that 62% of these were hotness or satiety stimuli, where no stimuli had RI scores above 0.4. Outside of the categories of hotness and satiety, only 12% of retained stimuli were categorised as poor or very poor based on RI scores.

#### Phase 2: Label selection and rating task

A total of 299 stimuli were selected based on the results of the free-labelling task. Of these stimuli, 191 vocalisations represented seven internal states (breathlessness, cold, fatigue, hotness, nausea, pain, and satiety) and 108 represented five control actions (chewing, humming, kissing, tongue clicking, and whistling). Participants were recruited from the SONA system at RHUL and via Prolific (www.prolific.com). Participants were required to have no hearing impairment, be aged 18 years or over, and be fluent in English. A total of 263 participants (88 male, 173 female, 2 other) aged 18–66 years (*M* = 25.01, *SD* = 8.66) took part in the rating task, delivered using Qualtrics (www.qualtrics.com). To reduce participant burden, each participant was asked to rate 100 stimuli, selected at random. On each trial, participants listened to a vocal stimulus as many times as they wished. Participants were presented with a list of the seven internal state and five control action labels (displayed in alphabetical order). Participants were asked to select which label(s) best described the vocalisation. For each label selected, participants were asked to rate how well it described the vocalisation, using a five-point Likert scale (*1 =* *very poorly, 2 = poorly, 3 = moderately, 4 = well, 5 = very well*). The task took approximately 30 minutes to complete.

#### Phase 2: Label selection and rating results

Each stimulus was rated by a mean of 83.2 participants (min = 65, max = 91). As the validity and quality of stimuli can be defined in different ways, and research studies will vary in their requirements, five stimulus measures were calculated, in line with those presented for the ISSI database (Biotti et al., 2022). The Quality Index (QI) is the mean rating given to the target (intended) label. A score of 0 was assigned whenever the target label was not selected. QIs range from 0 to 5 and reflect the extent to which the intended label is seen as a good descriptor of the vocalisation. The Specificity Index (SI) was calculated by subtracting the mean rating given to selected distractor labels from the rating given to the target label for each participant, and taking the mean of these values across all participants. SI scores are between −5 and 5, and reflect the extent to which the target label is perceived as a good descriptor of the vocalisation, over and above distractor states or control labels. The Maximum-Distractor Specificity Index (SI+) was obtained by subtracting the highest distractor rating from the target label rating, and taking the mean of these values across all participants. SI+ scores range between −5 and 5, and indicate the extent to which the target label is seen as a better descriptor than any other. The SI and SI+ are therefore more conservative scores of stimulus quality than the QI. The Choice Rate (CR) is the proportion of participants who selected the target label to describe the stimulus, regardless of the rating given. The High-Quality Choice Rate (CR+) is the proportion of participants who gave the target label the highest quality rating of all labels. CR and CR+ scores therefore range between 0 and 1, with CR+ being a more conservative estimate of stimulus recognisability. All five scores are presented for each stimulus in Appendix Table 5. See Biotti et al. (2022) for further discussion of these scores. Inter-rater agreement was estimated using intraclass correlation (consistency, using a two-way random-effects model, based on a mean rating (k = 261), calculated using R, which indicated a high level of consistency between raters in terms of ratings of the intended state/control label (ICC = .99, 95% CI [.99–.99]). All further statistical analyses were conducted using IBM SPSS version 25 software.

Across all five stimulus scores, control vocalisations received significantly higher scores than interoceptive state vocalisations (Table 1). It is worth noting that, while it may be possible to select difficulty-matched interoceptive and control stimuli based on these scores, the response options available to participants are likely to affect recognition accuracy. It is therefore also possible to increase or decrease recognition difficulty by adding or reducing the number of response options, or by selecting response options that are more or less likely to be confused with the intended response.

**Table 1:** T-tests comparing Quality Index (QI), Selectivity Index (SI), Maximum-Distractor Selectivity Index (SI+), Choice Rate (CR) and High-Quality Choice Rate (CR+) across the interoceptive state stimuli and control action stimuli

Stimulus score	Interoceptive State vocalisations mean (SD)	Control vocalisations mean (SD)	*t*-test for difference between means
QI	3.877 (.655)	4.316 (.381)	*t*(296.763) = 7.337, *p* < .001
SI	1.369 (2.316)	3.476 (1.044)	*t*(285.559) = −10.786,* p* < .001
SI+	1.230 (2.362)	3.423 (1.060)	*t*(285.204) = 11.016, *p* < .001
CR	0.717 (.274)	0.926 (.077)	*t*(238.458) = 9.896, *p* < .001
CR+	0.597 (.311)	0.875 (.115)	*t*(265.767) = 11.051, *p* < .001

**Fig. 1:**
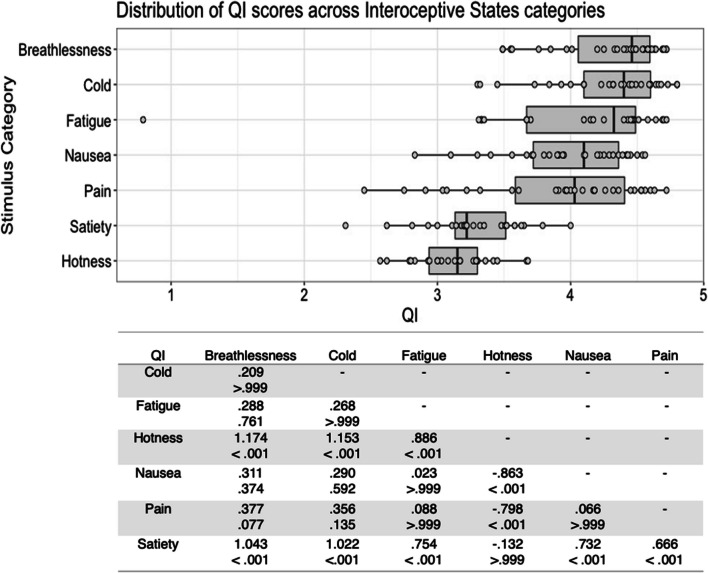
Box plots showing distribution of QI scores across all interoceptive states, with data points representing individual stimuli (top panel). Bottom panel shows results of Bonferroni-corrected *t*-tests indicating the difference in means (column – row) between each pair of internal states, with *t* values above *p* values in each cell

**Fig. 2:**
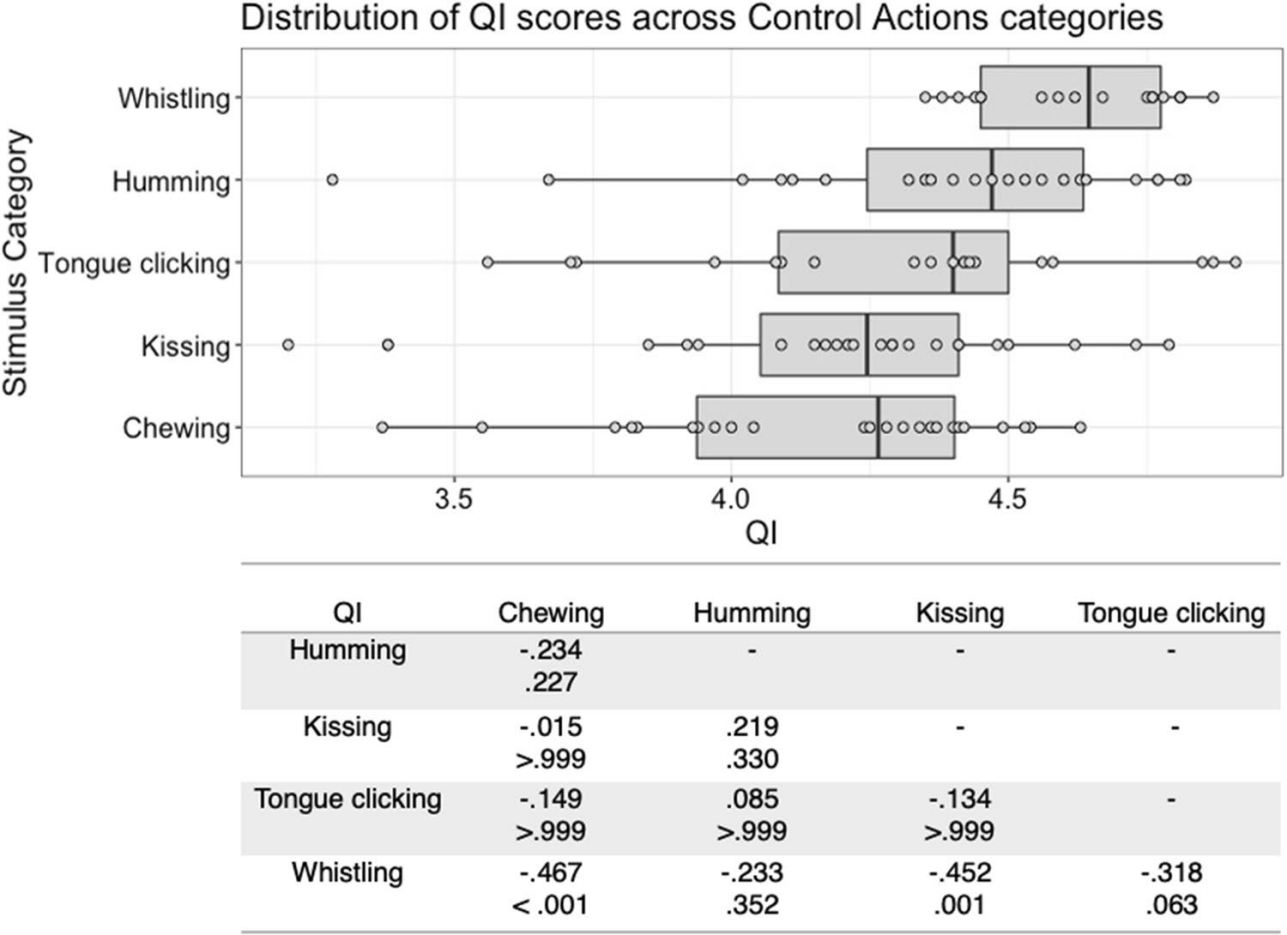
Box plots showing distribution of QI scores across all control actions, with data points representing individual stimuli (top panel). Bottom panel shows results of Bonferroni-corrected *t*-tests indicating the difference in means (column – row) between each pair of actions, with *t* values above *p* values in each cell

**Fig. 3:**
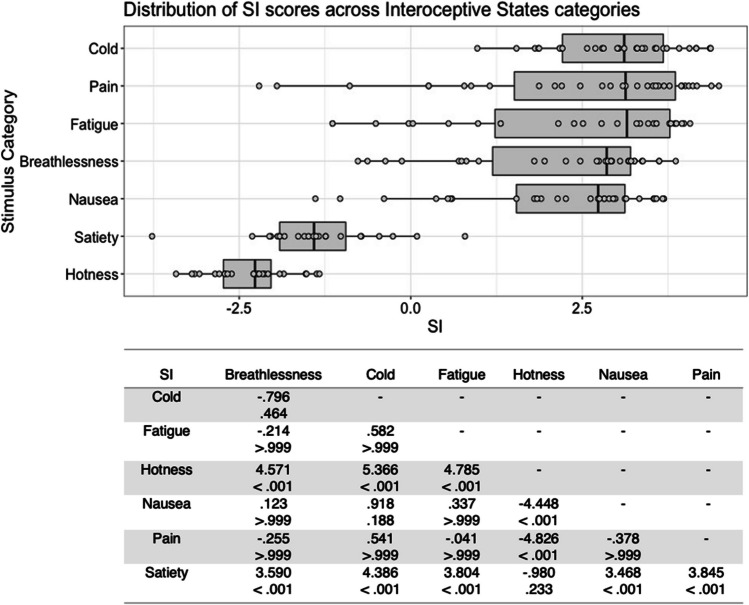
Box plots showing distribution of SI scores across all interoceptive states, with data points representing individual stimuli (top panel). Bottom panel shows results of Bonferroni-corrected *t*-tests indicating the difference in means (column – row) between each pair of internal states, with *t* values above *p* values in each cell

**Fig. 4:**
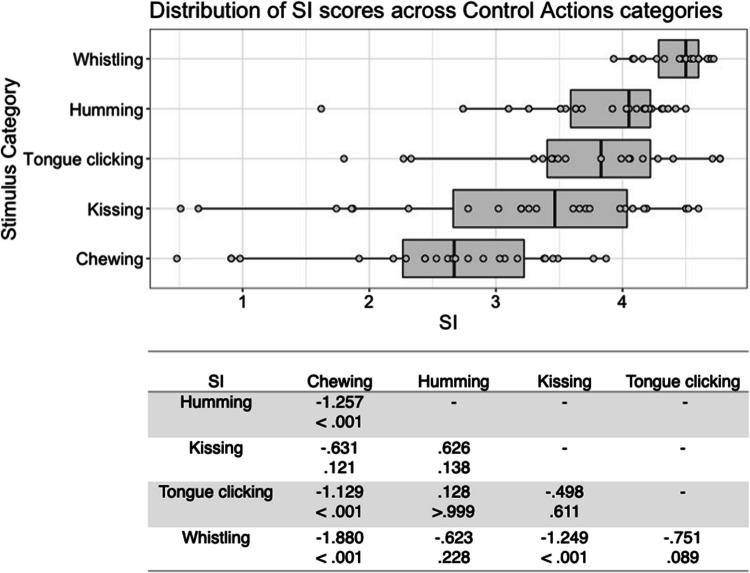
Box plots showing distribution of SI scores across all control actions, with data points representing individual stimuli (top panel). Bottom panel shows results of Bonferroni-corrected *t*-tests indicating the difference in means (column – row) between each pair of actions, with *t* values above *p* values in each cell

**Fig. 5:**
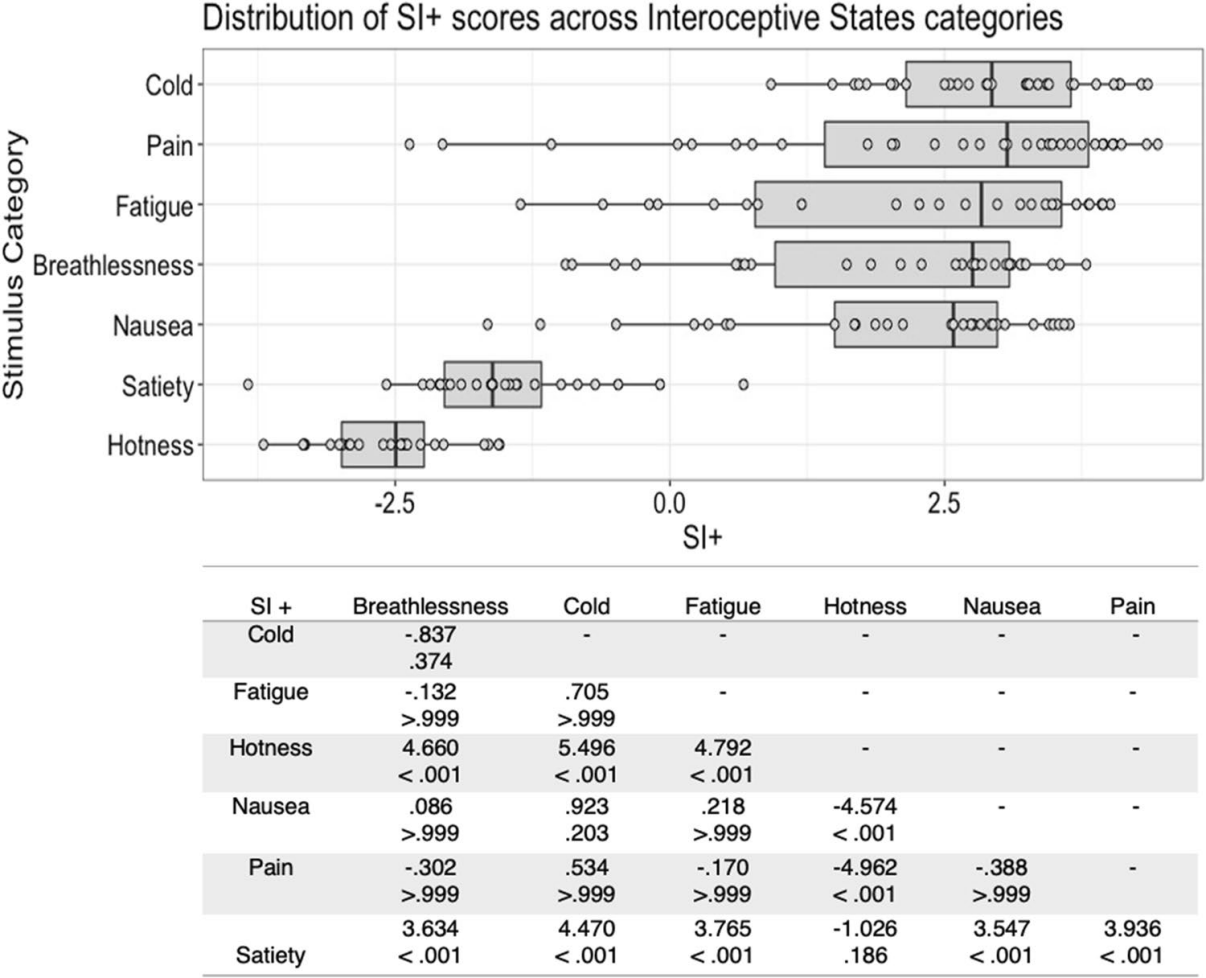
Box plots showing distribution of SI+ scores across all interoceptive states, with data points representing individual stimuli (top panel). Bottom panel shows results of Bonferroni-corrected *t*-tests indicating the difference in means (column – row) between each pair of internal states, with *t* values above *p* values in each cell

**Fig. 6:**
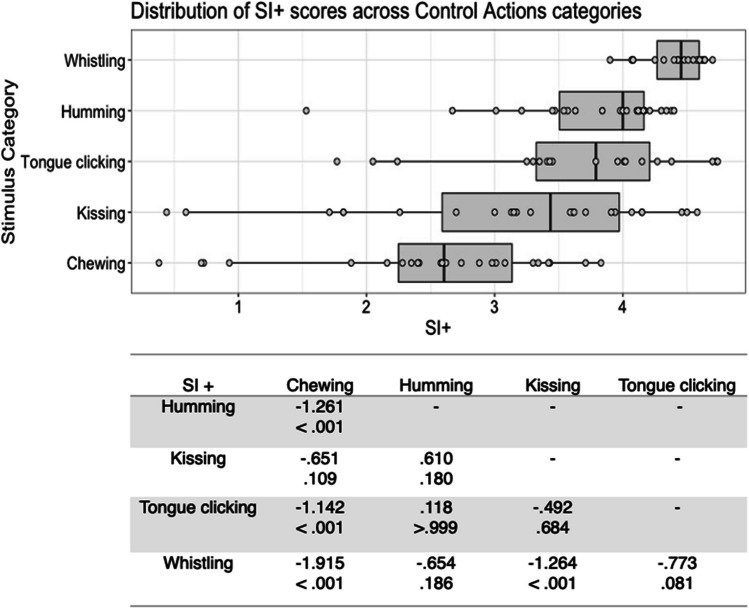
Box plots showing distribution of SI+ scores across all control actions, with data points representing individual stimuli (top panel). Bottom panel shows results of Bonferroni-corrected *t*-tests indicating the difference in means (column – row) between each pair of actions, with *t* values above *p* values in each cell

**Fig. 7:**
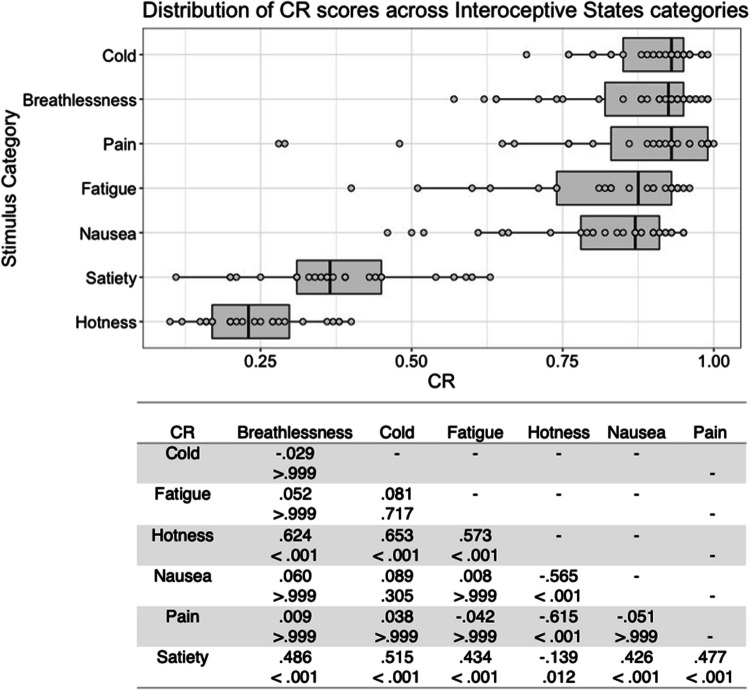
Box plots showing distribution of CR scores across all interoceptive states, with data points representing individual stimuli (top panel). Bottom panel shows results of Bonferroni-corrected *t*-tests indicating the difference in means (column – row) between each pair of internal states, with *t* values above *p* values in each cell

**Fig. 8:**
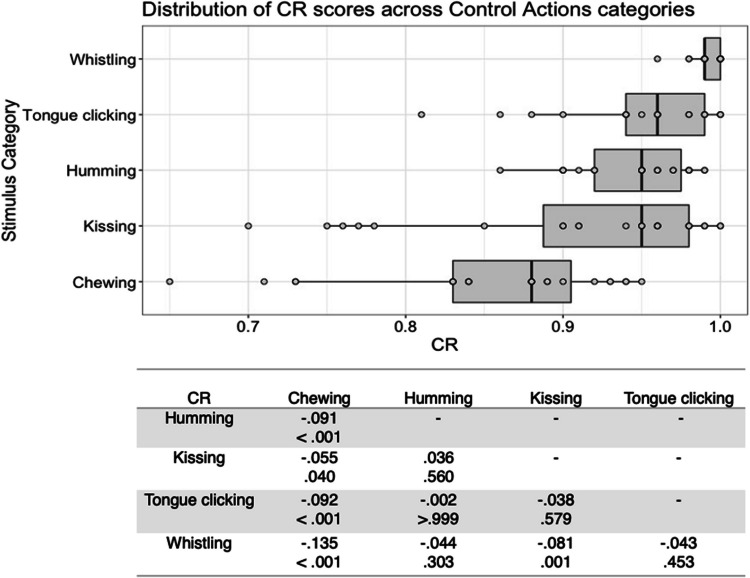
Box plots showing distribution of CR scores across all control actions, with data points representing individual stimuli (top panel). Bottom panel shows results of Bonferroni-corrected *t*-tests indicating the difference in means (column – row) between each pair of actions, with *t* values above *p* values in each cell

**Fig. 9:**
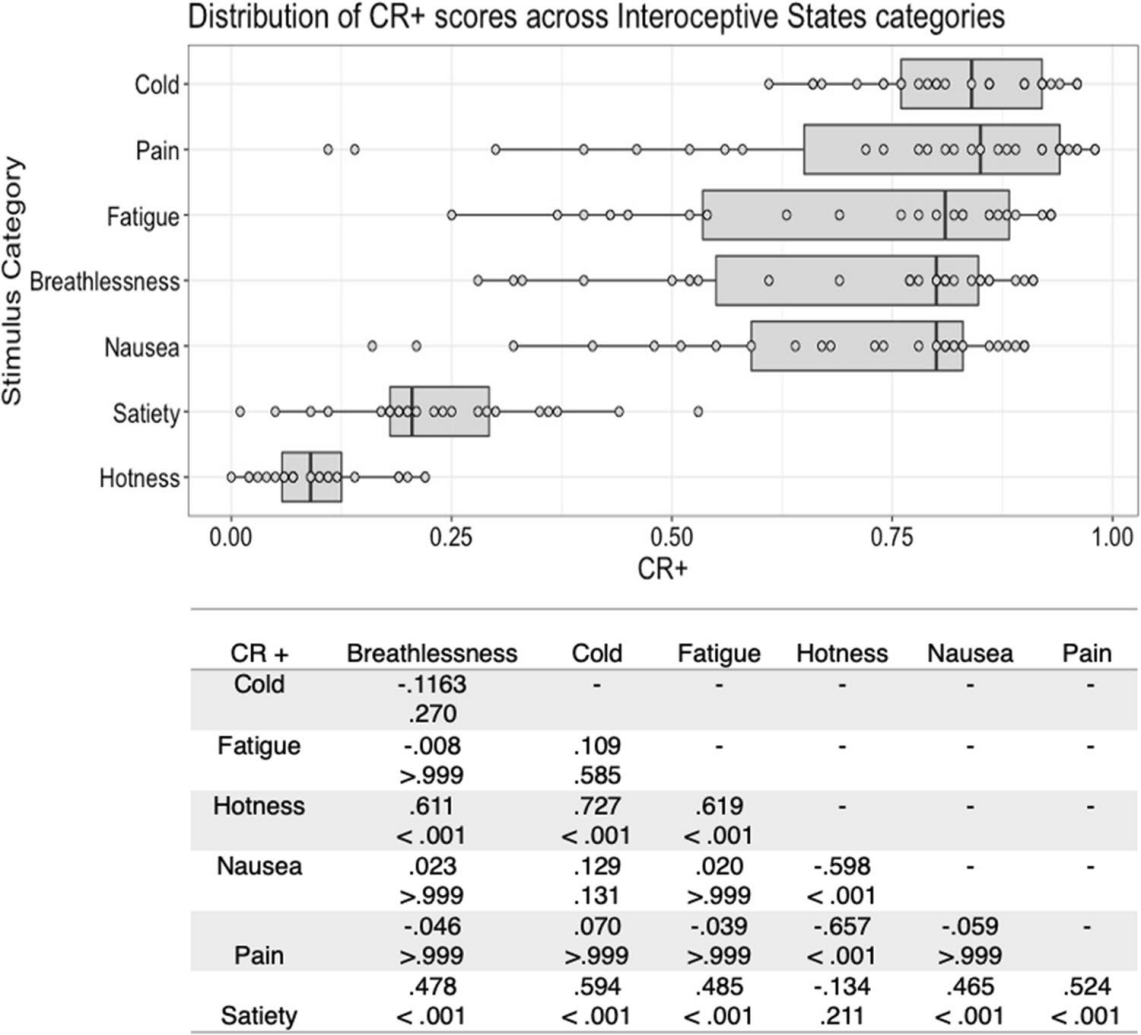
Box plots showing distribution of CR+ scores across all interoceptive states, with data points representing individual stimuli (top panel). Bottom panel shows results of Bonferroni-corrected *t*-tests indicating the difference in means (column – row) between each pair of internal states, with *t* values above *p* values in each cell

**Fig. 10:**
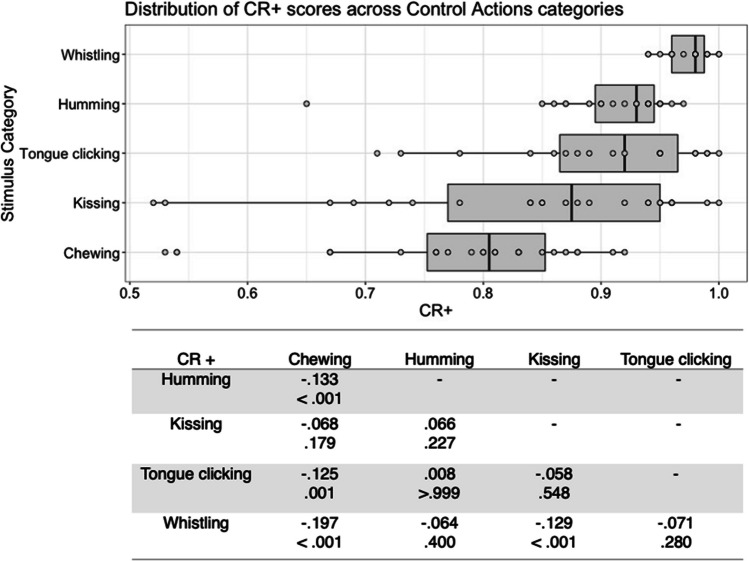
Box plots showing distribution of CR+ scores across all control actions, with data points representing individual stimuli (top panel). Bottom panel shows results of Bonferroni-corrected *t*-tests indicating the difference in means (column – row) between each pair of actions, with *t* values above *p* values in each cell

There was substantial variability in the five scores both within and between stimulus types (see Figs. 1, 2, 3, 4, 5, 6, 7, 8, 9, 10). Separate ANOVAs were conducted for interoceptive state and control action vocalisations, with the five stimulus scores as dependent variables, and stimulus type (all interoceptive state/control action stimulus categories) and actor sex (male, female) included as independent variables. Notably, the assumption of homogeneity of variance was violated for all five dependent variables, as recognisability was more varied among some stimulus categories than others. ANOVAs were conducted as this analysis is relatively robust to violations of this assumption, but Welch’s tests including the main independent variable of interest (stimulus type) were also conducted, and yielded the same pattern of significance as the ANOVAs (see Supplementary Materials).

For interoceptive state stimuli QI scores, there was a significant main effect of stimulus type, *F*(6, 177) = 22.473, *p* < .001, η^2^ = .432. Bonferroni corrected post hoc *t*-tests indicated that breathlessness stimuli were given the highest QI scores, but these only differed significantly from those for hotness and satiety, all *p* < .001. Stimuli in the hotness and satiety categories were given significantly lower QI scores than all other stimulus types, all *p* < .001 (see Fig. 1). While there was no significant main effect of actor sex on interoceptive state QI scores, *F*(1, 177) = 2.427, *p = *.121, η^2^ = .014, there was a significant interaction between stimulus type and actor sex, *F*(6, 177) = 2.194, *p = *.046, η^2^ = .069. Males and females did not differ significantly in QI, however, for any stimulus type; the interaction appears to be driven by the pattern of QI scores across the stimulus types varying between males and females, such as breathlessness stimuli receiving the highest QI for males, but the third highest for females, and fatigue stimuli receiving the second highest QI for female actors, but the fifth highest for male actors (see Figure S1, in Supplementary Materials).

For control action stimuli QI scores, there was a significant main effect of stimulus type, *F*(4, 98) = 6.125, *p* < .001, η^2^ = .200. Bonferroni corrected post hoc *t*-tests indicated that whistling had the highest QI, but this was only significantly higher than those for chewing and kissing, all *p* ≤ .001. Chewing was the stimulus type with the lowest QI, but this only differed significantly from whistling, *p* < .001 (Fig. 2). There was also a significant main effect of actor sex, *F*(1, 98) = 5.791, *p = *.018, η^2^ = .056, with female actors, *M* = 4.39 , *SD* = .333 , having a higher QI score than male actors, *M* = 4.23, *SD* = .413, but no significant interaction between stimulus type and actor sex, *F*(4, 98) = .134, *p = *.970, η^2^ = .005.

SI scores for the interoceptive state stimuli varied significantly with stimulus type, *F*(6, 177) = 65.845, *p* < .001, η^2^ = .691. Bonferroni corrected post hoc *t*-tests indicated that the stimulus type with the highest SI was cold, but this was only significantly higher than that for hotness and satiety, all *p* < .001. Stimuli expressing hotness and satiety were given significantly lower SI scores than all other stimulus types, all *p* < .001 (Fig. 3). There was no significant main effect for actor sex, *F*(1, 177) = .813, *p = *.368, η^2^ = .005, or interaction between actor sex and stimulus type, *F*(6, 177) = 1.433, *p = *.204, η^2^ = .046.

For the control action stimuli, there was a significant main effect of stimulus type on SI score, *F*(4, 98) = 14.325, *p* < .001, η^2^ = .369. Bonferroni corrected post hoc *t*-tests indicated that whistling was the stimulus type with the highest SI, differing significantly from chewing and kissing, all *p* < .001. Chewing stimuli were given the lowest SI, which differed significantly from all other stimuli except kissing, all *p* < .001 (Fig. 4). Neither a significant main effect of actor sex, *F*(1, 98) = 2.158, *p = *.145, η^2^ = .022, nor a significant interaction between actor sex and stimulus type, *F*(4, 98) = .341, *p = *.850, η^2^ = .014, was found for control stimuli SI.

There was a significant main effect of stimulus type, *F*(6, 177) = 66.312, *p* < .001, η^2^ = .692, on interoceptive stimuli SI+ score. Cold stimuli were associated with the highest SI+ scores, and Bonferroni corrected post hoc *t*-tests showed that these were significantly higher than for hotness and satiety stimuli, all *p* < .001. In line with the scores above, hotness and satiety stimuli were associated with significantly lower SI+ scores than all other stimulus types, all *p* < .001 (Fig. 5). There was no significant main effect of actor sex on SI+ score, *F*(1, 177) = 1.306, *p = *.255, η^2^ = .007, and no significant interaction between actor sex and stimulus type, *F*(6, 177) = 1.727, *p = *.117, η^2^ = .055.

For the control action stimuli, there was a significant main effect of stimulus type on SI+ score, *F*(4, 98) = 14.260, *p* < .001, η^2^ = .368. Again, whistling stimuli were found to have the highest SI+ scores, with Bonferroni corrected post hoc *t*-tests indicating that these differed significantly to those for chewing and kissing, all *p* < .001. Stimuli depicting chewing had lower SI+ scores than all stimulus types except kissing, all *p* < .001) (Fig. 6). There was no significant main effect of actor sex, *F*(1, 98) = 2.371, *p = *.127, η^2^ = .024, and no significant interaction between actor sex and stimulus type *F*(4, 98) = .379, *p = *.823, η^2^ = .015.

Where CR scores were concerned, for the interoceptive state stimuli there was a significant main effect of stimulus type, *F*(6, 177) = 95.753, *p* < .001, η^2^ = .764. Consistent with the previous scores, cold stimuli had the highest CR scores, but Bonferroni corrected post hoc *t*-tests found that these only differed significantly from hotness and satiety, all *p* < .001. Satiety stimuli received significantly lower CR scores than all other stimulus types except hotness, with hotness stimuli having significantly lower CR scores, all *p* < .012 (Fig. 7). Actor sex did not affect CR score for the interoceptive stimuli, *F*(1, 177) < .001, *p = *.993, η^2^ < .001, or interact with stimulus type, *F*(6, 177) = 1.347, *p = *.239, η^2^ = .044.

There was a significant main effect of stimulus type on CR score for the control action stimuli, *F*(4, 98) = 13.208, *p* < .001, η^2^ = .350. Bonferroni corrected post hoc *t*-tests found that whistling stimuli were significantly better recognised than chewing and kissing stimuli, all *p ≤ *.001, while chewing was significantly worse recognised than all other stimulus types, all *p ≤ *.040 (Fig. 8). There was no significant main effect of actor sex, *F*(1, 98) = .263, *p = *.609, η^2^ = .003, and no significant interaction between actor sex and stimulus type, *F*(4, 98) = .634, *p = *.640, η^2^ = .025.

Finally, there was a significant main effect of stimulus type on the CR+ score for internal state stimuli, *F*(6, 177) = 65.810, *p* < .001, η^2^ = .690. Bonferroni corrected post hoc *t*-tests indicated that cold stimuli had the highest CR+ scores, which were significantly higher than for hotness and satiety stimuli, all *p* < .001. Hotness and satiety stimuli had significantly lower CR+ scores than all other stimulus types, all *p <* .001 (Fig. 9). Actor sex did not significantly affect CR+ score, *F*(1, 177) = .282, *p = *.596, η^2^ = .002, or interact with stimulus type, *F*(6, 177) = 1.767, *p = *.108, η^2^ = .057.

For the control action stimuli, there was a significant main effect of stimulus type on CR+ scores, *F*(4, 98) = 12.328, *p* < .001, η^2^ = .335. Consistent with the four previously reported scores, whistling stimuli were highest in CR+, with Bonferroni corrected post hoc *t*-tests showing that this differed significantly from chewing and kissing CR+, all *p* < .001. Chewing stimuli had the lowest CR+ scores, differing significantly from all stimulus types except kissing, all *p ≤ *.001 (Fig. 10). There was no significant main effect of actor sex on CR + score, *F*(1, 98) = 2.042, *p = *.156, η^2^ = .020 and no significant interaction between actor sex and stimulus type *F*(4, 98) = .342, *p = *.849, η^2^ = .014.

Unsurprisingly, the five stimulus quality scores were significantly correlated with each other, for both interoceptive and control action stimuli, all *p* <.001 (see Tables 2 and 3), suggesting they are all valid measures of stimulus quality, despite assessing quality in different ways.

**Table 2:** Correlation matrix showing the significant positive relationships between the five stimulus scores for the interoceptive state vocalisation stimuli

	SI	SI +	CR	CR +
QI	.816 < .001	.848 < .001	.809 < .001	.839 < .001
SI	-	.996 < .001	.976 < .001	.990 < .001
SI +	-	-	.977 < .001	.995 < .001
CR	-	-	-	.977 < .001

**Table 3:** Correlation matrix showing the significant positive relationships between the five stimulus scores for the control action vocalisation stimuli

	SI	SI +	CR	CR +
QI	.869 < .001	.867 < .001	.740 < .001	.796 < .001
SI	-	.999 < .001	.958 < .001	.978 < .001
SI +	-	-	.956 < .001	.980 < .001
CR	-	-	-	.959 < .001

**Fig. 11:**
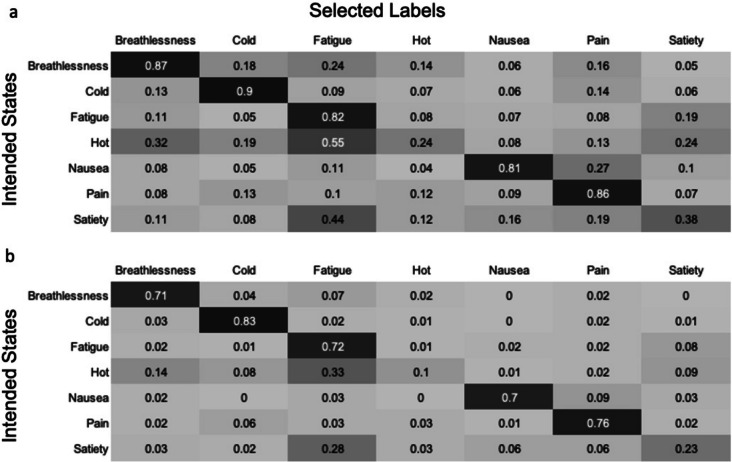
Confusion matrices showing the degree of confusion between interoceptive state vocalisations. Values show the mean proportion (taken across all stimuli within a given state category) of participants **a** selecting each label (CR), and **b** giving the highest rating to each label (CR+)

**Fig. 12:**
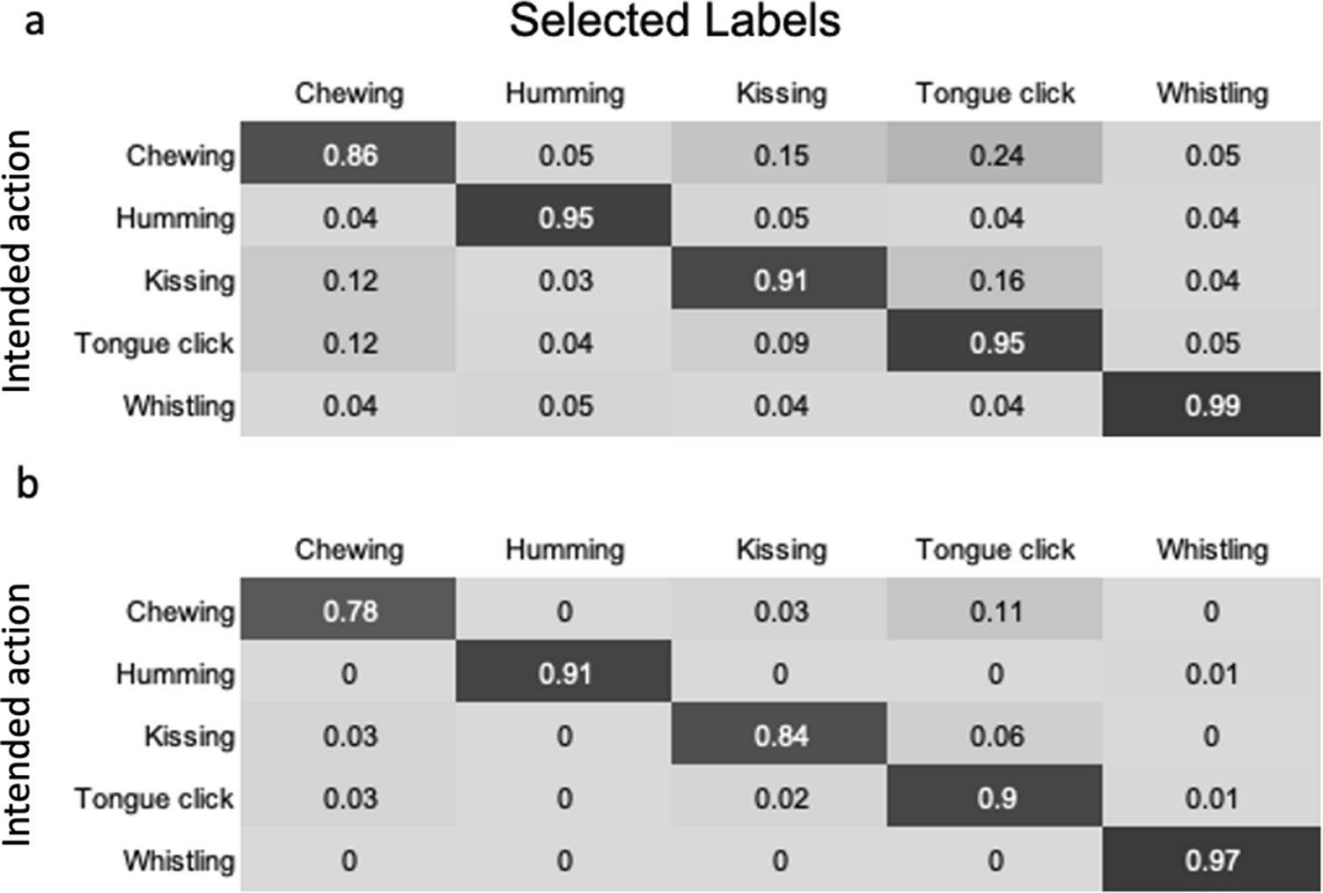
Confusion matrices showing the degree of confusion between control action vocalisations. Values show the mean proportion (taken across all stimuli within a given control action vocalisation category) of participants **a** selecting each label (CR), and **b** giving the highest rating to each label (CR+)

To quantify the extent to which stimuli were labelled as unintended states, and the degree of confusion between individual states, confusion matrices were produced, utilising CR and CR+ equations. For each interoceptive stimulus, the proportion of participants selecting each of the interoceptive state labels (CR) or giving each of the interoceptive state labels the highest rating (CR+) was calculated. The mean of these values was then calculated (Fig. 11). This process was repeated for the control action stimuli and all control action labels (Fig. 12). For the interoceptive state stimuli, confusion between intended and unintended states was generally low, although fatigue vocalisations were also frequently labelled as hotness, satiety and breathlessness, and satiety stimuli were often mislabelled as portraying hot and fatigue. The control action vocalisations were rarely mislabelled, but the vocalisations most likely to be confused with each other were chewing, tongue clicking and kissing.

## Interoceptive states point light displays database

### Point light display stimulus development

#### Actors

Ten adult actors (five male, five female), who were drama students at the University of Birmingham, were recruited to produce point light displays (PLDs). Actors gave informed consent for the PLDs resulting from recordings of their actions to be made available for use in research studies, shared with the scientific community, and presented at public talks and conferences. A financial remuneration was given to all actors for their time.

#### Procedure

**Fig. 13:**
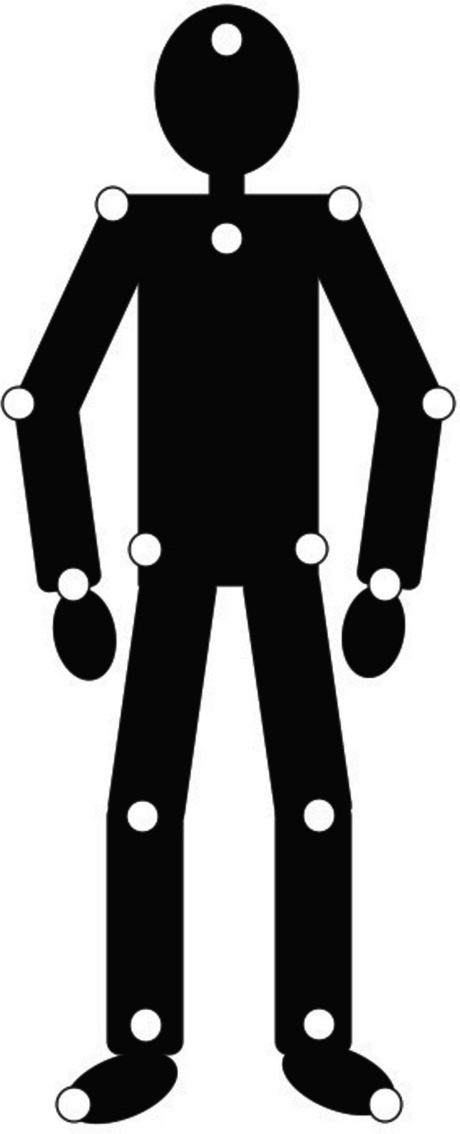
Schematic illustration of the locations of reflective markers attached to actors’ forehead and joints

Actors attended the recording laboratory at the University of Birmingham, where 16 12-mm-diameter reflective markers were attached to their skin or tightly fitting clothing at specific points of the body: forehead, shoulders, elbows, wrists, sternoclavicular joint (top of chest where the sternum and clavicle meet), hips, knees, ankles, toes (Fig. 13). The actors’ movements were recorded using 11 ceiling-mounted Qualisys Oqus 300 infrared cameras (Qualisys Inc., Stockholm), sampling at a rate of 120 Hz. The motion capture system was calibrated with a wand calibration tool, with each marker being tracked accurately to the nearest mm. Actors were asked to express ten interoceptive states (cold, fatigue, nausea, pain, breathlessness, hunger, thirst, hotness, satiety, and itch) seven times without any feedback, followed by three times with verbal direction from the experimenter, in an attempt to make the stimuli as recognisable as possible. Participants commenced each 15-second trial standing with their arms outstretched to the sides and legs together in a ‘T’ position, to allow accurate labelling of the reflective points by the Qualisys software. Actors did not produce control action PLDs, as these have previously been published. As PLDs contain very little identifying information (e.g. age, sex/gender, and physical characteristics are difficult to determine from PLDs), it is possible to use existing PLDs as control stimuli alongside the current PLDs.

#### Point light display stimulus editing

**Fig. 14:**
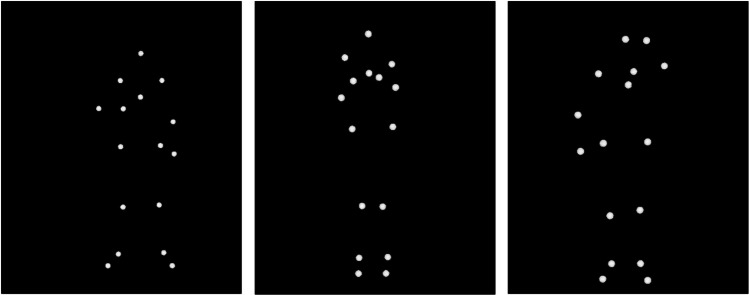
Still frames taken from point light display stimuli depicting breathlessness, cold, and hotness

Raw data files recorded via Qualisys were processed in MATLAB, where recording issues (e.g. body markers not displaying consistently) were resolved. A custom MATLAB script was used to resolve temporarily occluded or missing markers (up to 250 ms), primarily relying on MATLAB’s built-in spline interpolation function. For markers missing for more than 250 ms, however, spline interpolation was ineffective, and resulted in large trajectory reconstruction errors. To address this, an alternative technique was employed, based on visible neighbouring markers. For example, if a marker on the right shoulder was absent, its previously known position relative to other visible markers, such as the left shoulder, was used to temporarily create a virtual right-shoulder marker, thus overcoming gaps greater than 250 ms. Video files were then created and exported for each actor and stimulus type, by taking screen captures of the Qualisys recordings, using Screencast-O-Matic software (https://screenpal.com). These videos were then edited in Adobe Premier Pro, to create separate five second mp4 files for each exemplar of each interoceptive state. Examples of still frames from the point light display videos are shown in Fig. 14.

### Point light display stimulus validation

All videos went through a pre-selection process conducted by the researchers to ensure that the visual properties of each stimulus were high quality (e.g., videos were discarded if markers were not visible or flickered for substantial periods). Stimuli depicting ‘pain’ were removed due to low quality recordings for a high proportion of PLDs. Following the pre-selection process, a total of 159 stimuli with the highest visual quality were retained for validation.

#### Phase 1: Free-labelling task

Twenty-one students (20 female, one male) aged 18–21 years (*M* = 18.62, *SD* = .97) were recruited through the RHUL SONA system to take part in an online free-rating task (30 minutes duration), and received course credits for their participation. The task was designed using Gorilla Experiment Builder (www.gorilla.sc) (Anwyl-Irvine et al., 2020). The following written instructions were presented at the beginning of the task:

*You will be presented with one point light video at a time. For each one, you need to provide a very brief description of what you think the video represents (for example what the person is doing, thinking, or feeling). There will be many stimuli, so it’s very important that you keep your answers as brief as possible. Ideally, you will use a single word or a short phrase. For example, if you see a display depicting a person walking, you can simply answer ‘walking’. Do not spend too much time on each individual video. If you think that the video may represent multiple things, you can list them from the most likely to the least likely (e.g. ‘1. Walking; 2. Jogging’). If you are unsure about what the video represents enter a guess, but refrain from using phrases such as ‘I don’t know’. There are no right or wrong answers in this task. *The task took approximately 30 minutes to complete.

#### Phase 1: Free-labelling results

As with the vocalisation stimuli, participants’ free-labelling responses were coded by two independent researchers (SA, RQ) (‘1’ if the participant used the intended state label, or a semantically similar label, or label for an associated action, to describe the stimulus, and ‘0’ otherwise). Disagreements were resolved through discussion with a third researcher (FB), although inter-rater agreement was very high (*k* = .914). As for the vocalisation stimuli, recognisability index (RI; mean accuracy score across all participants) was calculated for each stimulus. The results indicated that, without guidance concerning the type of information being conveyed, participants found it challenging to interpret the stimuli (Appendix Table 6). The proportion of participants producing the intended label was very low (*M* = 22%, *SD* = 20%). Itch (*M* = 48%, *SD* = 17%, range 11–74%) and cold (*M* = 36%, *SD* = 18%, range 0–68%) were the best recognised states, while thirst (*M* <.01%, *SD* = 1%, range 0–5%) was the least well recognised state. It was assumed that the availability of response options would lead to more successful recognition of the stimuli (Russell, 1993); therefore, all 159 stimuli were retained for use in the second stage of validation.

#### Phase 2: Label selection and rating task

A total of 159 PLD videos used in the free-labelling validation stage were also rated in the second stage of validation, displaying nine internal states (breathlessness, cold, fatigue, hotness, hunger, itch, nausea, satiety, and thirst). Ninety-six participants aged 19–61 years (*M* = 28.83, *SD* = 8.98) were recruited via Prolific (www.prolific.com) and took part in the rating task (30 minutes duration). The task was designed using Gorilla Experiment Builder (Anwyl-Irvine et al., 2020). Participants were asked to rate all 159 PLD videos. For each trial, a video was presented as many times as the participant wished, accompanied by a list of the nine internal states (in alphabetical order). Participants were asked to select which label(s) best described the video. For each label selected, participants were asked to rate how well it described the video, using a five-point Likert scale (1=*very poorly*, 2=*poorly*, 3=*moderately*, 4=*well*, 5=*very well*).

#### Phase 2: Label selection and rating results

**Fig. 15:**
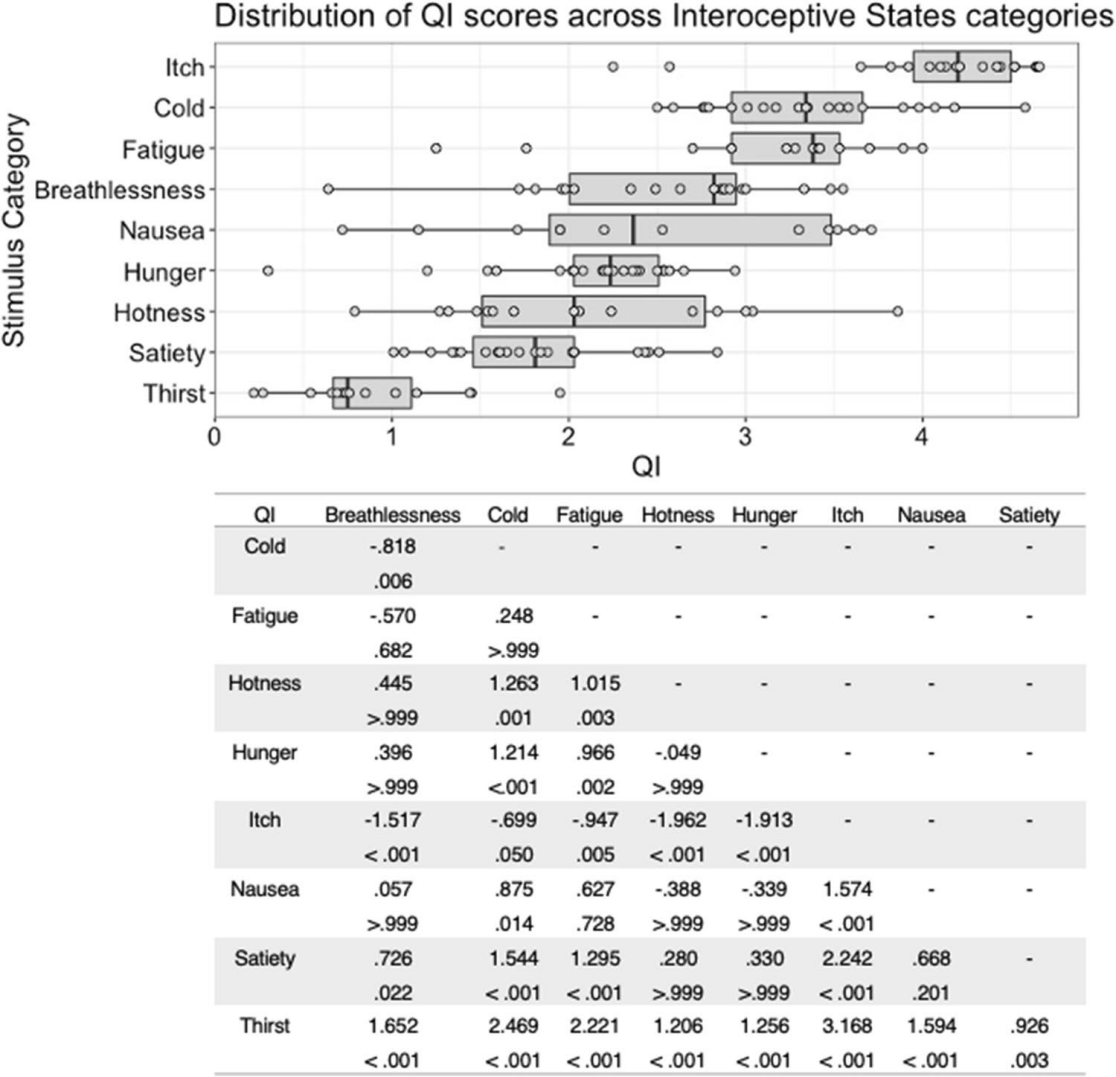
Box plots showing distribution of QI scores across all interoceptive states, with data points representing individual stimuli (top panel). Bottom panel shows results of Bonferroni-corrected *t*-tests indicating the difference in means (column – row) between each pair of internal states, with *t* values above *p* values in each cell

**Fig. 16:**
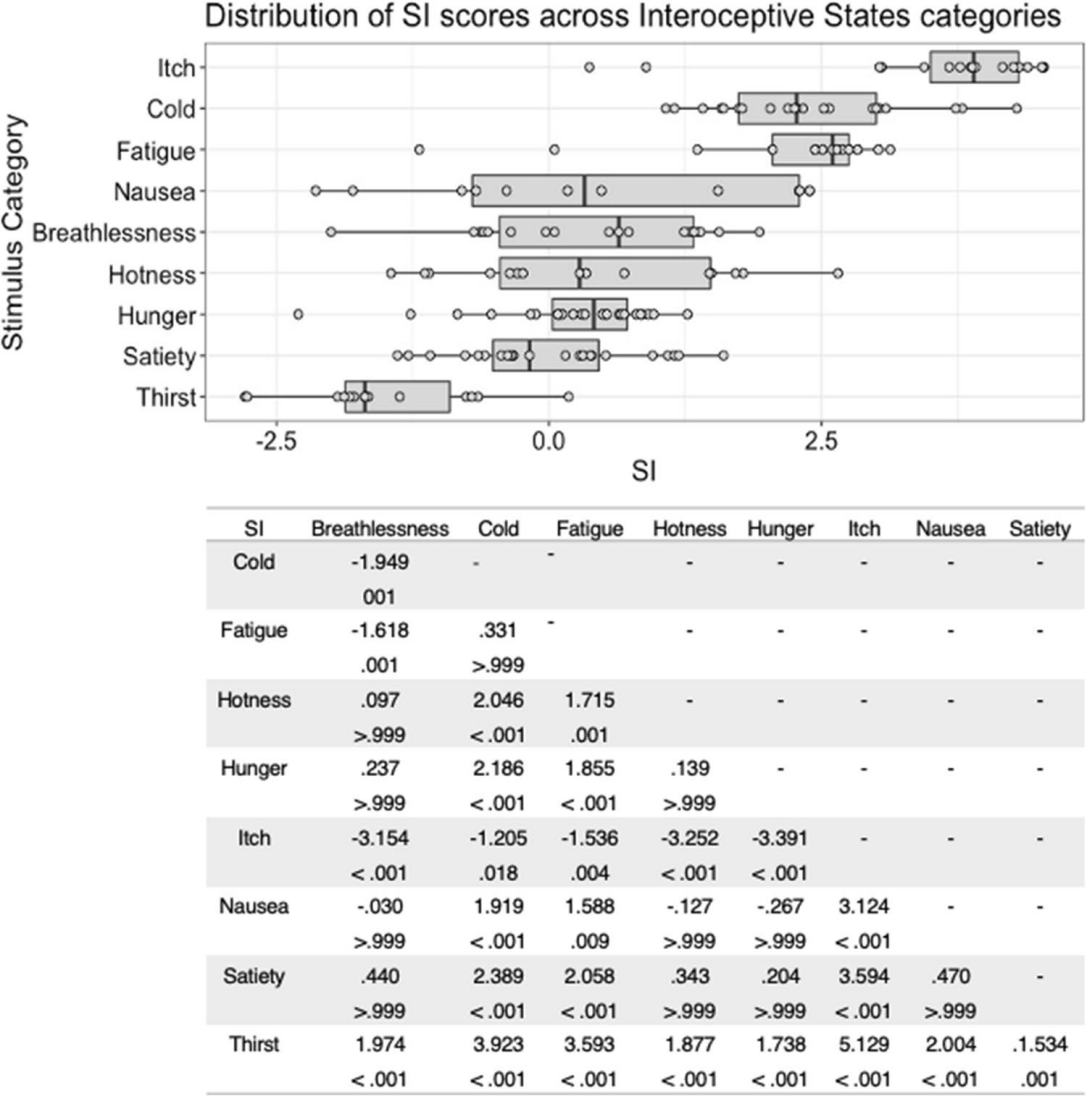
Box plots showing distribution of SI scores across all interoceptive states, with data points representing individual stimuli (top panel). Bottom panel shows results of Bonferroni-corrected *t*-tests indicating the difference in means (column – row) between each pair of internal states, with *t* values above *p* values in each cell

**Fig. 17:**
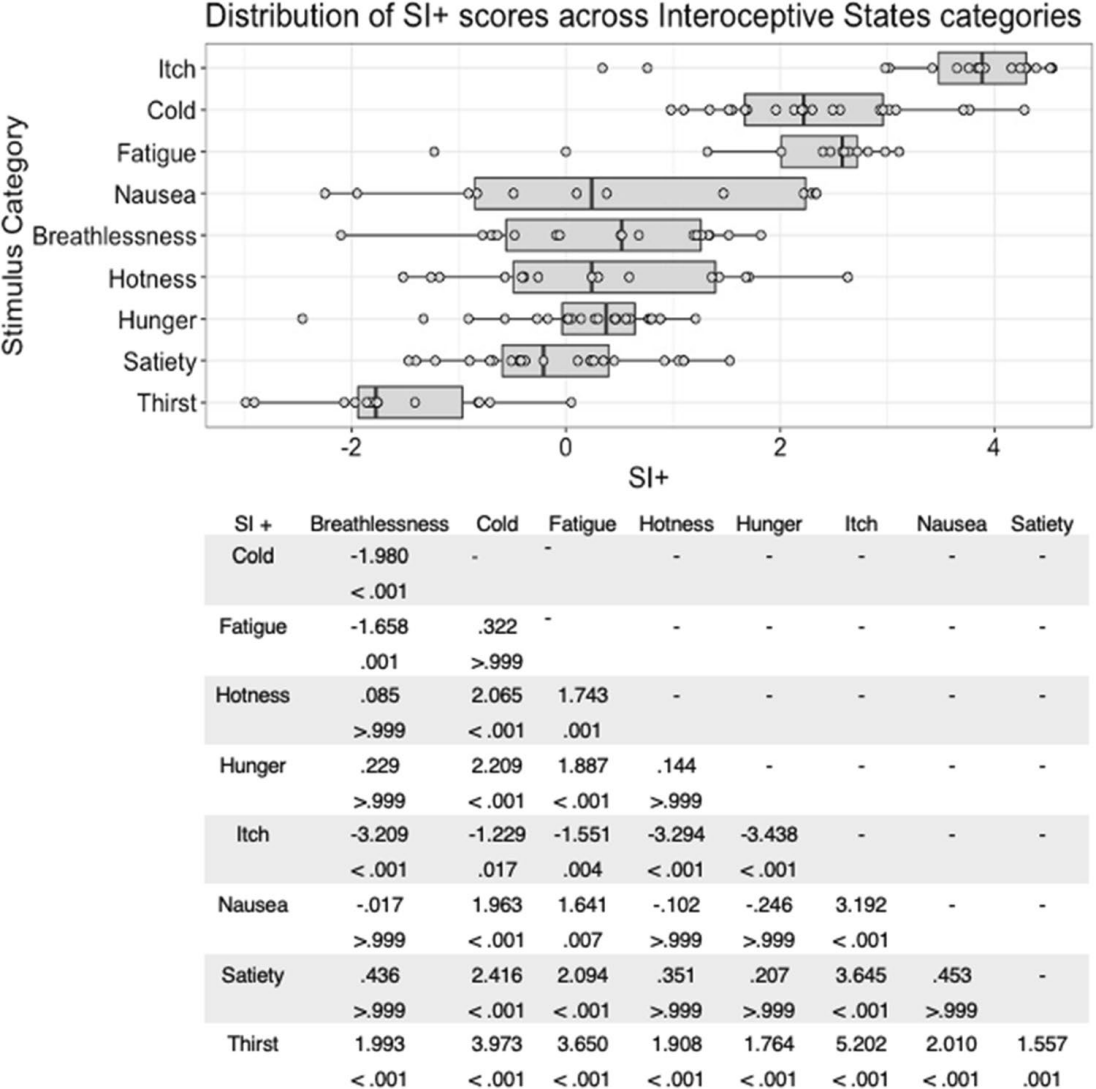
Box plots showing distribution of SI+ scores across all interoceptive states, with data points representing individual stimuli (top panel). Bottom panel shows results of Bonferroni-corrected *t*-tests indicating the difference in means (column – row) between each pair of internal states, with *t* values above *p* values in each cell

**Fig. 18:**
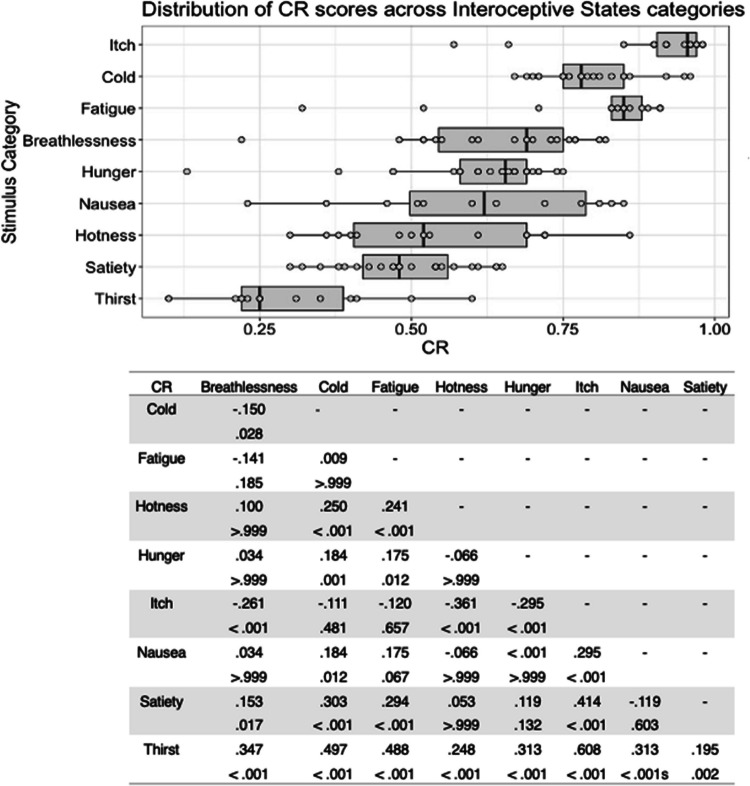
Box plots showing distribution of SI+ scores across all interoceptive states, with data points representing individual stimuli (top panel). Bottom panel shows results of Bonferroni-corrected *t*-tests indicating the difference in means (column – row) between each pair of internal states, with *t* values above *p* values in each cell

**Fig. 19:**
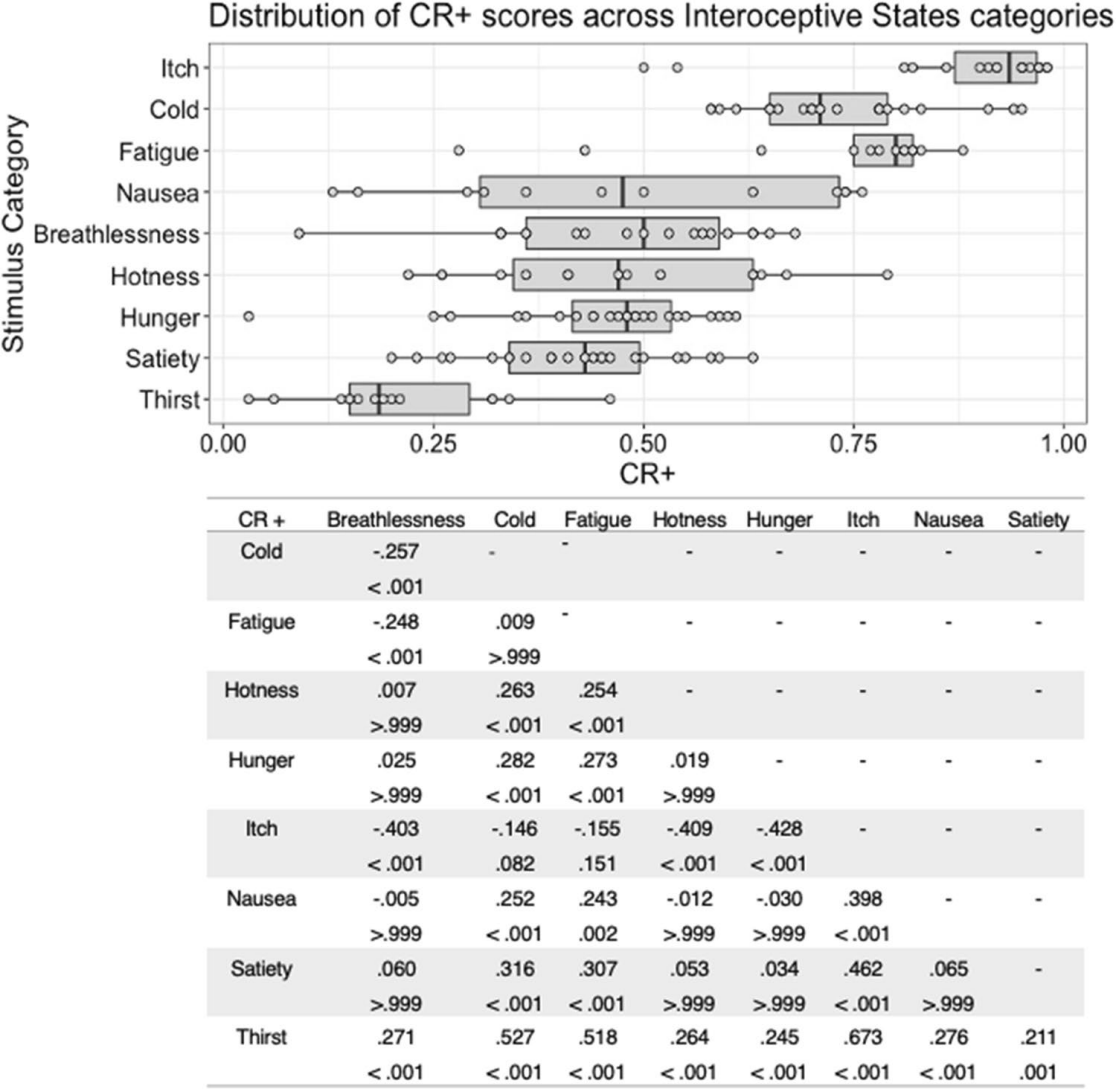
Box plots showing distribution of SI+ scores across all interoceptive states, with data points representing individual stimuli (top panel). Bottom panel shows results of Bonferroni-corrected *t*-tests indicating the difference in means (column – row) between each pair of internal states, with *t* values above *p* values in each cell

The quality and recognisability of PLD stimuli were measured using the scoring system previously implemented by Biotti et al. (2022) and as described above (QI, SI, SI+, CR, and CR+). All five scores for each stimulus are presented in Appendix Table 6. Over all stimuli, participants selected the intended state label the majority of the time (CR *M* = 64%, *SD* = 21%), but as with the vocalization stimuli, variability in the five scores was high both within and between stimulus types (see Figs. 15, 16, 17, 18, 19). For all five scores, we conducted an ANOVA with actor sex (male, female) × stimulus type (breathlessness, cold, fatigue, hotness, hunger, itch, nausea, satiety, thirst). For the PLD stimuli, the assumption of homogeneity of variance was violated for all five dependent variables, owing to recognition of stimuli being more variable within some stimulus categories than others. As with the ISV stimuli, ANOVA analyses were proceeded with as they are fairly robust to this violation, but Welch’s tests using stimulus type as the independent variable indicated the same pattern of significance (see Supplementary Materials). Inter-rater agreement was estimated using intraclass correlation (consistency, using a two-way random-effects model, based on a mean rating (k = 96), which indicated a high level of consistency between raters in terms of ratings of the intended state/control label (ICC = .94, 95% CI [.93–.96]). All statistical analyses were conducted using IBM SPSS version 25 software.

There was a significant main effect of stimulus type on QI scores, *F*(8, 141) = 31.91, *p* < .001, η^2^ = .644). Bonferroni corrected post hoc *t*-tests indicated that itch stimuli were given higher QI scores than all other stimuli, though this difference was at trend level for cold stimuli, *p* = .050, and significant for all remaining stimulus types (all *p* ≤ .005). QI scores were significantly lower for thirst stimuli than all other stimulus types, all *p* ≤ .003 (Fig. 15). Actor sex did not significantly affect QI score, *F*(1, 141) = .29, *p = *.592, η^2^ = .002, or interact with stimulus type, *F*(8, 141) = 1.29, *p = *.251, η^2^ = .068.

Where SI scores are concerned, there was a significant main effect of stimulus type, *F*(8, 141) = 35.25, *p* < .001, η^2^ = .667). As with QI scores, Bonferroni corrected post hoc *t*-tests indicated that itch stimuli were given higher ratings than all other stimulus types, all *p ≤ *.018, whilst thirst stimuli were associated with significantly lower SI scores than any other stimulus type, all *p ≤ *.001 (Fig. 16). There was no significant main effect of actor sex on SI scores, *F*(1, 141) = .72, *p = *.398, η^2^ = .005, and no significant interaction between actor sex and stimulus type, *F*(8, 141) = 1.39, *p = *.205, η^2^ = .073.

For SI+, there was a significant main effect of stimulus type, *F*(8, 141) = 35.24, *p* < .001, η^2^ = .667. Bonferroni corrected post hoc *t*-tests again found that SI+ scores were significantly higher for itch stimuli than other stimulus types, all *p ≤ *.017, and SI+ scores were significantly lower for thirst than other stimulus types, all *p ≤ *.001 (Fig. 17). There was neither a significant main effect of actor sex, *F*(1, 141) = .70, *p = *.403, η^2^ = .005, nor a significant interaction between actor sex and stimulus type, *F*(8, 141) = 1.42, *p = *.192, η^2^ = .075, when predicting SI+ scores.

CR score varied significantly with stimulus type, *F*(8, 141) = 27.68, *p* < .001, η^2^ = .611. Itch stimuli were again associated with the highest CR scores, and Bonferroni corrected post hoc *t*-tests showed that these were significantly higher than for all stimulus types except cold and fatigue (all *p* < .001). CR scores were again significantly lower for thirst stimuli than for all other stimulus types, all *p ≤ *.002 (Fig. 18). Actor sex did not significantly affect CR, as indicated by a non-significant main effect, *F*(1, 141) = .76, *p = *.386, η^2^ = .005, or interaction with stimulus type, *F*(8, 141) = 1.06, *p = *.396, η^2^ = .057.

For CR+, there was a significant main effect of stimulus type, *F*(8, 141) = 31.59, *p* < .001, η^2^ = .642. Bonferroni corrected post hoc *t*-tests demonstrated that, as with CR, itch stimuli had significantly higher CR+ scores than all stimulus types except cold and fatigue, all *p* < .001. As with all other stimulus scores, thirst stimuli had significantly lower CR+ than all other stimulus types, all *p ≤ *.001 (Fig. 19). There was no significant main effect of actor sex on CR+ ratings, *F*(1, 141) = .49, *p = *.485, η^2^ = .003), and no significant interaction between actor sex and stimulus type *F*(8, 141) = 1.27, *p = *.262, η^2^ = .067.

As with the vocalisation stimuli, the five stimulus quality scores were significantly correlated with each other, all *p* <.001 (Table 4).

**Table 4:** Correlation matrix showing the significant positive relationships between the five stimulus scores for the interoceptive state point light display stimuli

	SI	SI +	CR	CR +
QI	. 982 < .001	.982 < .001	.979 < .001	.975 < .001
SI	-	1.000 < .001	.963 < .001	.994 < .001
SI +	1.000 < .001	-	.962 < .001	.994 < .001
CR	-	-	-	.968 < .001

**Fig. 20:**
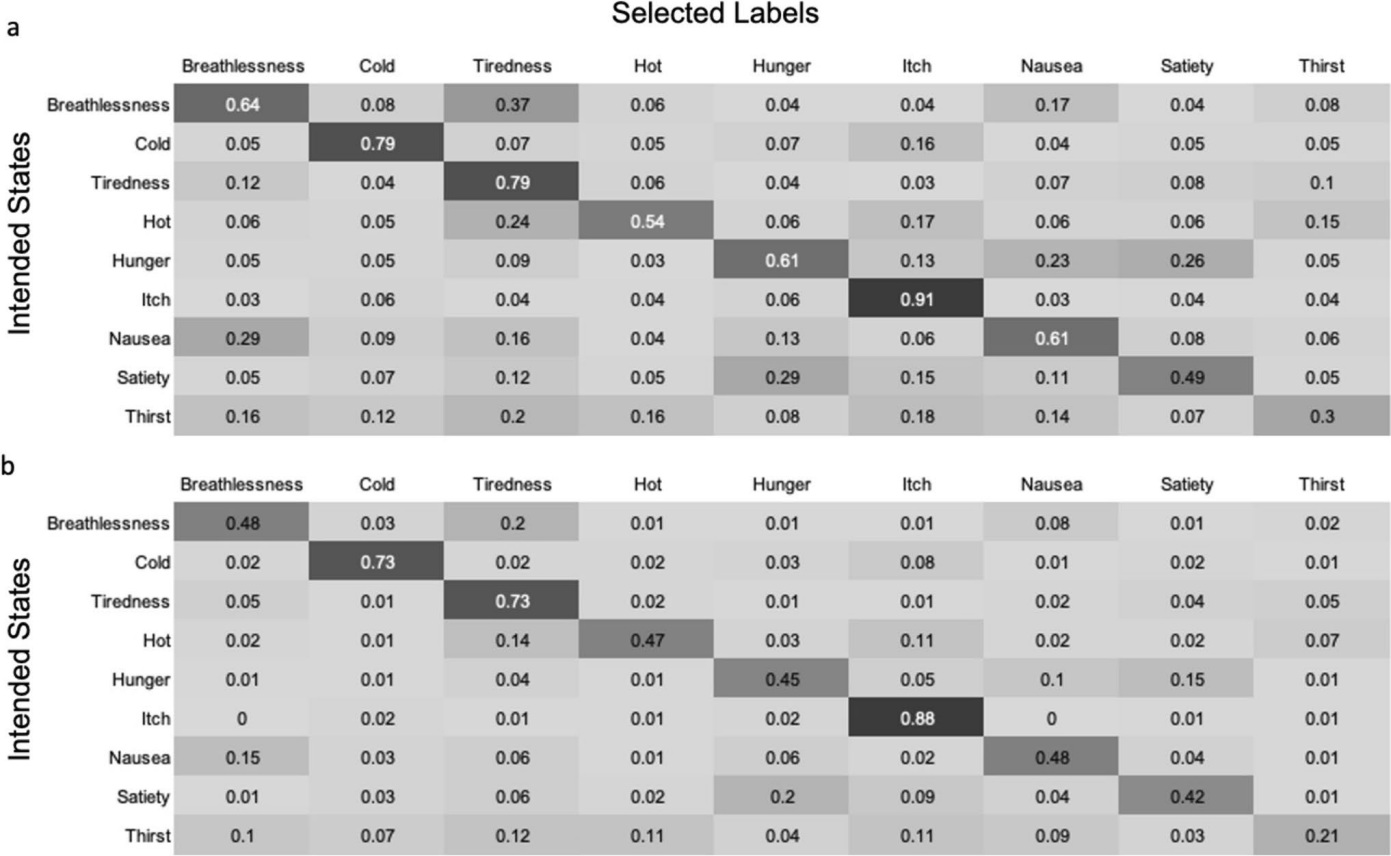
Confusion matrices showing the degree of confusion between interoceptive states. Values show the mean proportion (taken across all stimuli within a given state category) of participants **a** selecting each label (CR), and **b** giving the highest rating to each label (CR+)

As with the vocalisation stimulus set, confusion matrices were produced, using both CR and CR+ equations, to determine the extent to which each stimulus type was interpreted as portraying each of the interoceptive states (Fig. 20). Generally, rates of confusion with unintended states were low, although interoceptive states with lower stimulus scores were often interpreted as portraying other interoceptive states. For example, hotness stimuli were often mislabelled as fatigue (CR = 24%, CR+ = 14%) or thirst (CR = 15%, CR+ = 7%), while hunger stimuli were most commonly mislabelled as satiety (CR = 26%, CR+ = 15%). There was substantial variation in the degree of confusability as estimated by the CR and CR+ equations, for example, satiety being mislabelled as hunger (CR = 15%, CR+ = 2%), and hunger being mislabelled as nausea (CR = 23%, CR+ = 1%). When estimated with CR, thirst stimuli were more likely to be given unintended labels than the intended label, while this pattern was not observed for CR+ scores. The CR+ score is likely to reflect the state label that would have been selected in a forced choice task, as it indicates the proportion of participants giving the intended label a higher rating than any other label, so is arguably the more relevant for inferring confusability.

## Discussion

This paper describes the creation and validation of two stimulus sets, depicting a range of interoceptive states expressed vocally (Interoceptive States Vocalisations (ISV) database) and through kinematics, presented as point light displays (Interoceptive States Point Light Displays (ISPLD) database). The ISV database also includes control action vocalisations, such as whistling and tongue clicking. The stimuli presented here are available for use in research, allowing investigation of the processing and recognition of others’ interoceptive states across multiple modalities, and can be accessed free of charge through the Insulab website (https://www.insulab.uk).

The quality of the stimuli was assessed using five different scores, allowing it to be quantified according to different definitions of relevance to researchers. Within both the vocalisation and PLD stimulus sets, there was substantial variation in the scores both across and within stimulus types. Overall, a mean of 72% and 64% of participants selected the intended label for interoceptive states in the ISV and ISPLD, respectively. Control vocalisations in the ISV were even more likely to be assigned the intended label (93%). While the five quality scores assess slightly different aspects of stimulus quality, they were unsurprisingly strongly associated with each other. Interoceptive states with the highest scores on one variable also received the highest scores on others, suggesting that, although there is variability across individual stimuli, some interoceptive states are more recognisable than others, when expressed vocally or kinematically.

Some interoceptive state categories were recognised better than others. In the ISV, for example, cold stimuli tended to be recognised best, while those depicting hotness were consistently difficult to recognise. For ISPLD stimuli, itch was the best recognised state, and thirst the least well recognised state. It is likely that the better recognised states had both more distinctive expressions (e.g. vocalisations such as ‘brrr’ and shivering to portray feeling cold, or large repetitive arm motions to portray itch), leading to low confusability with other states. It is also possible that the frequency with which these expressions occur in daily life, and the extent to which they are associated with behavioural expressions, contributes to their recognisability. Scratching when one experiences an itch, and shivering when one is cold are likely to be common occurrences, as they are spontaneous responses (due to serving a purpose to restore a neutral state) to relatively common interoceptive signals. Thirst, on the other hand, is perhaps less strongly associated with behavioural responses other than taking action to relieve the state (i.e. drinking), and less frequently experienced as it is easier to pre-emptively avoid (by drinking small amounts throughout the day).

Interestingly, for interoceptive states that were included in both stimulus sets, those interoceptive states that were best recognised from motion cues were not necessarily the same states that were best recognised from vocalisation cues. For example, itch was consistently the best recognised state in the ISPLD set, but itch stimuli were removed from the validation procedure for the ISV database, as it was deemed too difficult to express vocally by actors. Similarly, breathlessness was one of the highest scoring states in the ISV, but was associated with more moderate scores in the ISPLD database. In comparison, some interoceptive states were recognised well across both stimulus sets, such as cold and fatigue, or do not appear to be conveyed well through either vocalisation or motion, such as hunger and thirst, which were removed from the vocalisation stimulus set after actors struggled to express them, and received low ratings across all five stimulus quality scores in the ISPLD set. The pattern of stimulus scores was more similar for the ISSI and ISPLD databases than for either with the ISV database, possibly due to the visual medium and postural cues present in both. Notably, the validation results from the three stimulus sets are not directly comparable, as validation was completed by different participants, and differences in the available labels may have altered interpretation of the stimuli. It is likely, however, that vocalisations and body postures and motion differ in the extent to which they are used to both express one’s own, and recognise others’, interoceptive states. This is potentially adaptive, as it may be possible to compensate for poor expression/recognition of a given state from cues in one modality, with more effective expression/recognition of cues in another modality. Similarly, ambiguous cues from one modality might be disambiguated by congruent cues from another modality, and where conflicting cues are present across modalities, cues may be weighted more heavily from one modality over another, depending on predictions concerning the interoceptive state. The existence of stimulus sets utilising information across different modalities is essential to determine how these cues to others’ interoceptive states are processed, making the combination of the ISPLD and ISV databases with the existing ISSI database (Biotti et al., 2022) crucial for research on social perception within the interoceptive domain.

To allow questions relating to the ambiguity of signals, or discrepancy across stimulus sets, to be investigated in future work, all stimuli that went through the second round of validation have been retained in the ISV and ISPLD databases. Researchers can therefore utilise the five stimulus quality scores to select the most appropriate stimuli for their own studies. Where stimuli are required that accurately portray the intended interoceptive state, we would recommend selecting those with high scores across the five stimulus quality measures. However, variability in scores will allow research designs involving ambiguous stimuli, or requirements for participants to sort or rate stimuli according to their recognisability. Multiple states overlap across the ISV, ISPLD, and ISSI, allowing for comprehensive research designs that have not previously been possible. Use of these stimulus sets in combination will allow investigation of the domain generality of the social perception of interoception, whether congruent or incongruent information from one modality biases perception of cues from another modality, the extent to which these cues are weighted, and whether this varies across individuals, for example as a function age, sex, or clinical diagnosis.

Use of PLDs in existing research has led to a number of interesting observations, such as sex differences in the processing of emotion (Alaerts et al., 2011), recognition of others’ emotion in autistic individuals (Actis-Grosso et al., 2015; Mazzoni et al., 2022), and sensitivity to sex differences in biological motion within the first year of life (Johnson et al., 2021). Similarly, research utilising vocalisations has investigated developmental, neurological, and clinically relevant aspects of vocal processing. Studies have investigated the developmental trajectories of vocal emotion recognition, and the neural bases underpinning these changes (Morningstar et al., 2018), observing improvements through childhood and adolescence, and decline in accuracy in late adulthood (Amorim et al., 2021). Other research groups have found that individuals with eating disorders (Kucharska-Pietura et al., 2004), depression (Kornreich et al., 2013), and autism spectrum conditions (Leung et al., 2022) often exhibit difficulties recognising emotion from others’ vocalisations, which may be explained by co-occurring alexithymia (Heaton et al., 2012). Relatedly, the ability to recognise one’s own emotions has been associated with recognition of others’ emotional states, often thought to suggest that we utilise our own emotional understanding (and perhaps emotion contagion) to infer others’ emotions (e.g. Bird & Viding, 2014; Grynberg et al., 2012). The current stimulus sets will allow investigation of whether recognition of others’ interoceptive states occurs simply through association of others’ expressive behaviours with verbal labels, or via our understanding of our own interoceptive states, or activation of these states within the self. It is clear from the scope of this work, and growing interest in emotional cues from vocal and kinematic information, that the availability of vocal and PLD stimuli depicting interoceptive states has the potential to facilitate varied research studies, combining the fields of social perception and interoception. Indeed, the interoceptive states represented in the ISV and ISPLD share many features with states traditionally described as ‘emotions’; they can be plotted within the Circumplex Model of affect (Russell, 1980), in that they could be described in terms of their valence and arousal level (Feldman et al., 2024), and they are likely to be inferred based on both physiological and contextual cues, as in Schachter and Singer’s model of emotion (Schachter & Singer, 1962). While these interoceptive states have not traditionally been considered to be emotions in the existing literature, the availability of the current stimuli presents the opportunity to investigate similarities and differences between these states and both basic and complex emotions.

Notably, interoceptive state stimuli tended to score less highly on the stimulus quality indices than control action vocalisation stimuli. It is possible that this is due to the high level of perceptual similarity between interoceptive state stimuli in terms of their associated expressive cues, while the control vocalisations are less perceptually similar to each other, leading to lower confusability amongst the control categories. Satiety, hotness, and fatigue, for example, were often vocalised using sighs, with the resulting perceptual similarities likely giving rise to the high levels of confusion between these states. As higher numbers of plausible response options tend to be associated with lower recognition accuracy (Russell, 1993), more confusability among the interoceptive states categories may have contributed to lower scores on the quality indices. As perceptual similarity/distinctiveness of stimuli (within and between categories) and the number of plausible response options are both likely to affect recognition accuracy, researchers should consider these factors and carefully select stimuli and response options when designing their studies.

The current stimuli were created by actors posing the interoceptive states, in line with the majority of emotional expression stimulus sets, including those utilising vocalisations and PLDs (e.g. Alaerts et al., 2011; Bidet-Ildei et al., 2020; Biotti & Cook, 2016; Cowen et al., 2019; Lima et al., 2013; Lorey et al., 2012; Mazzoni et al., 2022; Simon-Thomas et al., 2009; Sowden et al., 2021). While these stimuli may vary from expressions elicited more automatically in response to experiencing an interoceptive state, as has been found for facial emotional expressions (Schmidt et al., 2006; Valstar et al., 2006), it is likely that individuals use body posture and motion, and vocalisations, to deliberately inform others of their internal state. For example, vocalisations such as ‘brrr’ to indicate that one is cold may be more likely in social situations than when one is alone, indicating a communicative role of interoceptive vocalisations. In contrast, scratching in response to itch may be a spontaneous behaviour that is inhibited in some social situations. Further work is therefore required to distinguish between expression and recognition of posed and spontaneous vocalisations and body postures and movement, and determine whether these vary as a function of interoceptive state. However, the development of stimuli depicting naturally induced interoceptive states, which could be utilised alongside the current stimuli, is required.

In conclusion, the ISV and ISPLD databases present a wide range of interoceptive vocalisations and PLDs, as well as control action vocalisations, which are freely available for use in research studies. It is anticipated that these stimuli will allow investigation of the correlates of recognition of others’ interoceptive states, including from neurobiological, developmental, and clinical perspectives, across multiple modalities. Multiple definitions of stimulus quality are presented alongside stimuli, allowing researchers to select the most appropriate stimuli for their purposes. Similarly, variability in stimulus quality has been retained in the databases, offering more and less ambiguous stimuli to be utilised where appropriate. Researchers are encouraged to report their rationale for selecting stimuli transparently, to aid with the interpretation of findings.

## Electronic supplementary material

Below is the link to the electronic supplementary material.

Supplementary file1 (DOCX 77 KB)

## Appendix 1

**Table 5:** Table showing stimulus recognisability and quality scores for each individual stimulus in the Interoceptive States Vocalisations (ISV) database. Recognisability Index (RI) was obtained in Validation Phase One (Free Labelling Task). Quality Index (QI), Selectivity Index (SI), Maximum Distractor Selectivity Index (SI+), Choice Rate (CR) and High-Quality Choice Rate (CR+) were obtained in Validation Phase Two (Label Selection and Rating Task). Actor ID includes the actor number (A1-A10) followed by their sex (M = male, F = female)

Stimulus	Stimulus type	Actor ID	RI	QI	SI	SI+	CR	CR+
1	Breathlessness	A1M	0.87	4.25	2.74	2.66	0.93	0.81
2	Breathlessness	A1M	0.87	4.42	2.84	2.76	0.92	0.8
3	Breathlessness	A1M	0.73	4.55	3.26	3.21	0.94	0.84
4	Breathlessness	A2M	0.67	4.4	2.26	2.1	0.88	0.77
5	Breathlessness	A2M	0.67	4.54	2.91	2.75	0.94	0.8
6	Breathlessness	A2M	0.73	4.01	0.99	0.74	0.81	0.5
7	Breathlessness	A3M	0.93	4.63	3.17	3.11	0.95	0.86
8	Breathlessness	A3M	0.67	4.72	3.26	3.06	0.95	0.81
9	Breathlessness	A3M	0.73	4.69	3.86	3.79	0.99	0.89
10	Breathlessness	A4M	0.27	3.49	-0.77	-0.95	0.64	0.33
11	Breathlessness	A4M	0.4	3.76	-0.13	-0.31	0.64	0.4
12	Breathlessness	A5M	0.67	3.85	0.7	0.63	0.71	0.52
13	Breathlessness	A5M	0.93	4.64	3.62	3.55	0.97	0.91
14	Breathlessness	A6M	0.93	4.6	2.47	2.29	0.91	0.77
15	Breathlessness	A6M	0.6	3.55	-0.63	-0.89	0.57	0.28
16	Breathlessness	A6M	0.93	4.58	2.71	2.6	0.93	0.82
17	Breathlessness	A7F	0.27	3.49	-0.37	-0.5	0.62	0.32
18	Breathlessness	A7F	0.27	3.56	0.81	0.68	0.74	0.52
19	Breathlessness	A8F	0.93	4.45	3.37	3.19	0.98	0.85
20	Breathlessness	A8F	0.8	4.33	2.87	2.79	0.96	0.85
21	Breathlessness	A8F	0.73	4.72	3.62	3.48	0.99	0.91
22	Breathlessness	A9F	0.33	4.7	3.38	3.24	0.97	0.9
23	Breathlessness	A9F	0.8	4.61	2.93	2.78	0.93	0.8
24	Breathlessness	A10F	0.87	4.47	3.18	3.09	0.96	0.86
25	Breathlessness	A10F	0.8	4.49	2.92	2.84	0.88	0.78
26	Breathlessness	A11F	0.33	4.49	3.05	2.96	0.94	0.77
27	Breathlessness	A11F	0.73	4.58	3.21	3.09	0.91	0.81
28	Breathlessness	A12F	0.67	4.2	1.8	1.61	0.85	0.61
29	Breathlessness	A12F	0.87	3.97	0.74	0.6	0.75	0.53
30	Breathlessness	A12F	0.87	4.35	1.95	1.83	0.89	0.69
31	Cold	A1M	0.53	3.3	1.54	1.48	0.76	0.71
32	Cold	A1M	0.6	3.32	0.97	0.92	0.69	0.61
33	Cold	A2M	0.6	4.4	3.11	2.93	0.93	0.8
34	Cold	A2M	0.47	4.29	2.79	2.62	0.92	0.76
35	Cold	A2M	0.67	4.1	1.81	1.68	0.85	0.66
36	Cold	A3M	0.73	4.64	3.03	2.88	0.93	0.86
37	Cold	A3M	0.53	4.49	2.19	2.04	0.83	0.67
38	Cold	A3M	0.53	4.6	3.41	3.35	0.93	0.9
39	Cold	A4M	0.67	3.45	1.86	1.79	0.8	0.74
40	Cold	A4M	0.53	3.73	2.17	2.01	0.89	0.79
41	Cold	A5M	0.67	4.73	3.01	2.89	0.88	0.8
42	Cold	A5M	0.87	4.45	3.68	3.65	0.94	0.93
43	Cold	A6M	0.8	4.38	3.56	3.43	0.96	0.9
44	Cold	A6M	1	4.44	3.37	3.24	0.96	0.84
45	Cold	A7F	0.87	3.93	2.69	2.55	0.9	0.81
46	Cold	A7F	0.73	4	2.21	2.15	0.8	0.74
47	Cold	A8F	0.93	4.32	3.29	3.25	0.93	0.86
48	Cold	A8F	0.53	4.23	3.3	3.27	0.93	0.9
49	Cold	A8F	0.93	4.53	3.73	3.68	0.96	0.92
50	Cold	A9F	0.53	4.1	2.57	2.5	0.83	0.78
51	Cold	A9F	0.67	3.84	1.87	1.72	0.76	0.66
52	Cold	A10F	0.93	4.46	4.16	4.09	0.98	0.96
53	Cold	A10F	0.87	4.66	3.58	3.45	0.94	0.86
54	Cold	A10F	0.87	4.6	3.91	3.88	0.95	0.92
55	Cold	A11F	0.53	4.32	2.81	2.72	0.91	0.76
56	Cold	A11F	0.73	4.59	4.16	4.1	0.96	0.94
57	Cold	A12F	0.87	4.8	4.36	4.29	0.98	0.96
58	Cold	A12F	0.93	4.68	4.06	4.04	0.95	0.92
59	Cold	A12F	0.87	4.8	4.37	4.35	0.99	0.96
60	Fatigue	A1M	0.33	4.15	2.15	2.06	0.82	0.69
61	Fatigue	A1M	0.33	4.25	2.51	2.45	0.83	0.78
62	Fatigue	A2M	0.2	3.33	-0.03	-0.19	0.63	0.37
63	Fatigue	A2M	0.33	3.7	1.31	1.2	0.83	0.63
64	Fatigue	A3M	0.4	3.33	0.03	-0.11	0.6	0.43
65	Fatigue	A3M	0.27	3.35	0.55	0.4	0.71	0.45
66	Fatigue	A4M	0.4	0.79	3.77	0.7	0.74	0.54
67	Fatigue	A4M	0.4	3.67	0.98	0.8	0.74	0.52
68	Fatigue	A5M	1	4.48	3.54	3.52	0.92	0.89
69	Fatigue	A5M	0.73	4.17	2.38	2.27	0.81	0.76
70	Fatigue	A6M	0.73	4.58	3.34	3.29	0.9	0.83
71	Fatigue	A6M	0.93	4.43	3.29	3.19	0.9	0.83
72	Fatigue	A7F	0.67	4.51	3.85	3.81	0.94	0.93
73	Fatigue	A7F	1	4.1	2.78	2.69	0.86	0.8
74	Fatigue	A8F	1	4.47	3.87	3.82	0.94	0.88
75	Fatigue	A8F	1	4.46	3.98	3.93	0.96	0.93
76	Fatigue	A9F	1	4.64	3.79	3.7	0.94	0.93
77	Fatigue	A9F	0.87	4.69	3.46	3.42	0.89	0.86
78	Fatigue	A10F	0.07	3.31	-1.14	-1.36	0.4	0.25
79	Fatigue	A10F	0.47	3.67	-0.51	-0.61	0.51	0.4
80	Fatigue	A11F	1	4.45	3.58	3.48	0.93	0.87
81	Fatigue	A11F	1	4.4	3.01	2.98	0.93	0.82
82	Fatigue	A12F	1	4.7	4.07	4.01	0.95	0.93
83	Fatigue	A12F	1	4.72	3.95	3.94	0.93	0.92
84	Hot	A1M	0	2.93	-2.61	-2.83	0.17	0.05
85	Hot	A1M	0	2.57	-2.22	-2.44	0.24	0.09
86	Hot	A2M	0	3.13	-2.25	-2.54	0.28	0.07
87	Hot	A2M	0	3.03	-1.85	-2.06	0.37	0.19
88	Hot	A3M	0	2.83	-2.13	-2.43	0.29	0.09
89	Hot	A3M	0.07	3.27	-1.91	-2.14	0.32	0.14
90	Hot	A4M	0	3.42	-3.15	-3.32	0.15	0.02
91	Hot	A4M	0	3.3	-3.19	-3.33	0.12	0.02
92	Hot	A5M	0	3	-2.71	-2.98	0.2	0.06
93	Hot	A5M	0	3.08	-2.71	-2.92	0.16	0.1
94	Hot	A6M	0	2.62	-3.42	-3.7	0.1	0
95	Hot	A6M	0	2.79	-3.08	-3.34	0.16	0.03
96	Hot	A7F	0.13	3.45	-1.37	-1.55	0.38	0.22
97	Hot	A7F	0	3.17	-2.16	-2.4	0.27	0.11
98	Hot	A8F	0.07	3	-2.08	-2.27	0.2	0.12
99	Hot	A8F	0.27	2.8	-2.21	-2.39	0.25	0.1
100	Hot	A9F	0	3.3	-1.33	-1.56	0.38	0.19
101	Hot	A9F	0	3.17	-1.53	-1.65	0.36	0.2
102	Hot	A10F	0	2.94	-2.85	-3.09	0.21	0.07
103	Hot	A10F	0	3.67	-2.67	-2.91	0.22	0.07
104	Hot	A11F	0	3.29	-2.79	-3.01	0.2	0.04
105	Hot	A11F	0	3.29	-2.29	-2.45	0.17	0.06
106	Hot	A12F	0	3.68	-2.29	-2.61	0.27	0.11
107	Hot	A12F	0	3.36	-1.52	-1.69	0.4	0.22
108	Nausea	A1M	0.67	3.71	2.62	2.56	0.85	0.81
109	Nausea	A1M	0.87	3.91	2.82	2.76	0.88	0.83
110	Nausea	A2M	0.27	4.36	3.33	3.31	0.93	0.89
111	Nausea	A2M	0.33	4.42	2.82	2.74	0.88	0.82
112	Nausea	A3M	0.73	4.54	3.54	3.45	0.92	0.87
113	Nausea	A3M	0.8	3.95	1.9	1.87	0.8	0.68
114	Nausea	A4M	0.6	4.25	2.73	2.67	0.9	0.81
115	Nausea	A4M	0.8	3.72	1.8	1.69	0.78	0.64
116	Nausea	A4M	0.8	3.8	0.6	0.51	0.61	0.51
117	Nausea	A5M	0.87	4.56	3.69	3.64	0.93	0.9
118	Nausea	A5M	0.73	4.45	3.12	2.98	0.9	0.86
119	Nausea	A5M	0.53	3.4	0.58	0.55	0.73	0.55
120	Nausea	A6M	0.6	3.94	2.26	2.12	0.84	0.74
121	Nausea	A6M	0.67	3.9	1.54	1.5	0.79	0.59
122	Nausea	A7F	0.47	3.3	0.37	0.22	0.66	0.41
123	Nausea	A7F	0.73	3.1	-0.39	-0.49	0.46	0.32
124	Nausea	A8F	0.87	4.29	3.55	3.49	0.95	0.9
125	Nausea	A8F	0.93	4.22	2.98	2.92	0.93	0.83
126	Nausea	A9F	0.87	4.43	3.58	3.54	0.95	0.9
127	Nausea	A9F	0.53	4.32	2.89	2.83	0.91	0.8
128	Nausea	A9F	0.8	4.5	3.67	3.59	0.92	0.88
129	Nausea	A10F	0.4	3.56	-1.39	-1.66	0.52	0.16
130	Nausea	A10F	0.53	3.84	2.14	1.98	0.82	0.73
131	Nausea	A11F	0.73	4.2	3.14	3.05	0.9	0.82
132	Nausea	A11F	0.8	4.39	2.95	2.94	0.87	0.81
133	Nausea	A11F	0.73	4.1	1.84	1.68	0.8	0.67
134	Nausea	A12F	0.8	3.67	0.54	0.35	0.65	0.48
135	Nausea	A12F	0.87	4.11	2.75	2.58	0.87	0.78
136	Nausea	A12F	0.47	2.83	-1.03	-1.18	0.5	0.21
137	Pain	A1M	0.53	3.04	0.26	0.07	0.67	0.4
138	Pain	A1M	0.27	2.45	-0.89	-1.075	0.48	0.3
139	Pain	A2M	0.53	3.32	1.15	1.02	0.8	0.58
140	Pain	A2M	0.53	3.61	0.78	0.6	0.76	0.52
141	Pain	A2M	0.73	3.56	0.88	0.75	0.76	0.56
142	Pain	A3M	0.8	3.97	2.2	2.05	0.91	0.74
143	Pain	A3M	0.67	4	2.1	2.02	0.86	0.78
144	Pain	A3M	0.67	3.22	1.87	1.8	0.89	0.72
145	Pain	A4M	0.53	3.96	3.61	3.56	0.99	0.94
146	Pain	A4M	1	4.72	4.49	4.44	1	0.98
147	Pain	A4M	1	4.6	4.38	4.34	0.99	0.98
148	Pain	A5M	0.8	4.17	3.45	3.38	0.99	0.92
149	Pain	A5M	0.8	4.56	3.69	3.65	0.96	0.89
150	Pain	A5M	0.73	4.26	3.3	3.25	0.94	0.88
151	Pain	A6M	0.6	4.48	4	3.95	0.99	0.94
152	Pain	A6M	1	4.45	4.05	4.04	0.99	0.95
153	Pain	A6M	0.67	4.63	4.11	4.03	0.98	0.94
154	Pain	A7F	0	2.75	-1.95	-2.07	0.29	0.14
155	Pain	A7F	0.07	2.91	-2.21	-2.37	0.28	0.11
156	Pain	A8F	0.27	3.07	0.26	0.2	0.65	0.46
157	Pain	A8F	0.93	4.18	3.95	3.94	0.99	0.96
158	Pain	A9F	0.33	4.53	3.94	3.87	0.99	0.92
159	Pain	A9F	0.27	3.91	2.91	2.82	0.93	0.81
160	Pain	A10F	0.87	4.53	4.17	4.11	0.99	0.96
161	Pain	A10F	0.33	4.03	3.53	3.45	0.99	0.94
162	Pain	A11F	0.8	3.97	2.79	2.67	0.91	0.82
163	Pain	A11F	0.8	4.36	3.57	3.48	0.99	0.87
164	Pain	A11F	0.8	3.89	2.47	2.41	0.9	0.79
165	Pain	A12F	0.73	4.09	3.13	3.07	0.93	0.85
166	Pain	A12F	0.8	4.18	3.09	3.04	0.92	0.84
167	Pain	A12F	0.87	4.32	3.77	3.75	0.96	0.94
168	Satiety	A1M	0	3.2	-1.55	-1.76	0.33	0.2
169	Satiety	A1M	0.07	2.81	-2.04	-2.25	0.2	0.09
170	Satiety	A2M	0.13	3.48	0.09	-0.09	0.59	0.44
171	Satiety	A2M	0.07	3.18	-0.26	-0.47	0.6	0.37
172	Satiety	A3M	0	3.65	-0.72	-0.99	0.57	0.3
173	Satiety	A3M	0	3.27	-1.64	-1.9	0.31	0.18
174	Satiety	A4M	0.2	3.79	0.79	0.67	0.63	0.53
175	Satiety	A4M	0.27	3.63	-0.46	-0.68	0.43	0.35
176	Satiety	A5M	0.2	3.58	-1.02	-1.23	0.45	0.29
177	Satiety	A5M	0.2	3.2	-1.49	-1.63	0.37	0.25
178	Satiety	A6M	0.07	3.22	-3.77	-3.84	0.11	0.01
179	Satiety	A6M	0.07	3.32	-0.73	-0.84	0.54	0.36
180	Satiety	A7F	0	3.11	-1.4	-1.61	0.36	0.18
181	Satiety	A7F	0.07	4	-1.24	-1.39	0.25	0.2
182	Satiety	A8F	0	3.51	-1.24	-1.4	0.45	0.23
183	Satiety	A8F	0.67	3.52	-1.91	-2.1	0.34	0.19
184	Satiety	A9F	0	3.14	-1.42	-1.62	0.44	0.17
185	Satiety	A9F	0.07	2.62	-2.31	-2.58	0.2	0.05
186	Satiety	A10F	0	2.31	-1.94	-2.04	0.21	0.11
187	Satiety	A10F	0.2	3.21	-2.06	-2.18	0.35	0.21
188	Satiety	A11F	0.13	3	-1.91	-2.09	0.31	0.19
189	Satiety	A11F	0.27	2.93	-1.84	-2	0.36	0.18
190	Satiety	A12F	0.2	3.22	-1.34	-1.46	0.39	0.28
191	Satiety	A12F	0.27	3.35	-1.38	-1.5	0.39	0.24
192	Chewing	A1M	0.6	3.97	2.62	2.58	0.88	0.81
193	Chewing	A1M	0.73	4.54	3.49	3.43	0.93	0.88
194	Chewing	A1M	0.73	4.4	3.07	3.01	0.88	0.81
195	Chewing	A2M	0.8	3.37	2.44	2.41	0.88	0.83
196	Chewing	A2M	0.87	3.94	2.66	2.62	0.88	0.8
197	Chewing	A2M	0.73	3.93	2.9	2.88	0.9	0.85
198	Chewing	A3M	0.6	4.49	3.77	3.71	0.95	0.91
199	Chewing	A3M	0.73	4.28	3.17	3.08	0.94	0.83
200	Chewing	A3M	0.6	4.24	2.68	2.59	0.89	0.8
201	Chewing	A4M	0.67	4	0.91	0.73	0.71	0.53
202	Chewing	A4M	0.6	3.83	1.92	1.88	0.83	0.67
203	Chewing	A4M	0.53	3.79	0.91	0.71	0.73	0.54
204	Chewing	A7F	0.73	4.04	2.19	2.16	0.84	0.76
205	Chewing	A7F	0.6	4.36	2.78	2.74	0.84	0.79
206	Chewing	A7F	0.67	4.53	3.45	3.42	0.88	0.86
207	Chewing	A8F	0.8	4.63	3.87	3.83	0.94	0.92
208	Chewing	A8F	0.6	4.41	3.38	3.3	0.93	0.88
209	Chewing	A8F	0.67	4.42	3.03	2.98	0.89	0.83
210	Chewing	A9F	0.67	4.25	2.44	2.35	0.83	0.77
211	Chewing	A9F	0.47	4.37	3.39	3.34	0.92	0.87
212	Chewing	A9F	0.47	4.31	2.29	2.28	0.83	0.76
213	Chewing	A10F	0.67	4.34	2.53	2.4	0.84	0.73
214	Chewing	A10F	0.6	3.55	0.98	0.93	0.73	0.67
215	Chewing	A10F	0.6	3.82	0.48	0.38	0.65	0.54
216	Humming	A1M	0.27	3.28	1.62	1.53	0.86	0.65
217	Humming	A1M	0.53	3.67	2.74	2.67	0.9	0.85
218	Humming	A2M	0.53	4.44	4.05	3.98	0.99	0.95
219	Humming	A2M	0.67	4.5	4.17	4.11	0.96	0.94
220	Humming	A2M	0.6	4.47	4.03	4	0.96	0.92
221	Humming	A3M	0.53	4.63	4.42	4.38	0.99	0.96
222	Humming	A3M	0.53	4.82	4.31	4.21	0.97	0.94
223	Humming	A3M	0.53	4.53	4.11	4.03	0.95	0.93
224	Humming	A4M	0.53	4.35	3.55	3.47	0.92	0.87
225	Humming	A4M	0.67	4.64	4.23	4.17	0.98	0.95
226	Humming	A4M	0.6	4.32	3.92	3.84	0.96	0.94
227	Humming	A7F	0.73	4.4	3.63	3.63	0.92	0.91
228	Humming	A7F	0.8	4.17	3.68	3.57	0.98	0.9
229	Humming	A7F	0.67	4.09	3.26	3.21	0.9	0.86
230	Humming	A8F	0.67	4.77	4.5	4.4	0.98	0.94
231	Humming	A8F	0.67	4.77	4.32	4.3	0.97	0.97
232	Humming	A8F	0.6	4.81	4.36	4.34	0.95	0.95
233	Humming	A9F	0.67	4.6	4.18	4.13	0.95	0.94
234	Humming	A9F	0.8	4.73	4.21	4.16	0.95	0.93
235	Humming	A9F	0.6	4.56	4.18	4.12	0.98	0.95
236	Humming	A10F	0.47	4.02	3.1	3.01	0.91	0.87
237	Humming	A10F	0.47	4.36	3.51	3.45	0.9	0.89
238	Humming	A10F	0.47	4.11	3.63	3.54	0.95	0.9
239	Kissing	A1M	1	4.79	4.5	4.46	0.98	0.96
240	Kissing	A1M	0.87	4.62	4.52	4.5	1	1
241	Kissing	A1M	1	4.41	4.08	4.07	0.98	0.96
242	Kissing	A2M	0.87	4.21	3.2	3.13	0.9	0.84
243	Kissing	A2M	1	4.27	3.2	3.17	0.91	0.84
244	Kissing	A2M	1	4.29	3.71	3.61	0.96	0.89
245	Kissing	A3M	0.6	3.38	1.87	1.82	0.76	0.69
246	Kissing	A3M	0.6	3.38	0.65	0.59	0.7	0.53
247	Kissing	A3M	0.47	3.2	0.51	0.44	0.75	0.52
248	Kissing	A4M	0.6	4.09	3.26	3.15	0.94	0.85
249	Kissing	A4M	0.93	4.32	3.61	3.59	0.96	0.94
250	Kissing	A4M	0.87	4.19	3.32	3.28	0.91	0.88
251	Kissing	A7F	1	4.73	4.6	4.58	1	0.99
252	Kissing	A7F	1	4.5	4.19	4.15	0.98	0.94
253	Kissing	A7F	0.93	4.48	4.17	4.15	0.96	0.96
254	Kissing	A8F	0.73	3.92	3.02	3	0.95	0.85
255	Kissing	A8F	1	4.22	4.02	3.94	0.99	0.95
256	Kissing	A8F	0.67	4.41	3.66	3.62	0.95	0.87
257	Kissing	A9F	0.6	3.85	1.74	1.71	0.78	0.72
258	Kissing	A9F	0.6	4.17	2.31	2.26	0.85	0.74
259	Kissing	A9F	0.73	3.94	1.86	1.82	0.77	0.67
260	Kissing	A10F	0.87	4.15	2.78	2.7	0.9	0.78
261	Kissing	A10F	1	4.37	3.98	3.91	0.99	0.95
262	Kissing	A10F	0.87	4.29	3.74	3.71	0.96	0.92
263	Tongue clicking	A1M	0.47	3.72	2.27	2.05	0.86	0.73
264	Tongue clicking	A1M	0.33	4.15	3.37	3.25	0.94	0.86
265	Tongue clicking	A2M	0.67	4.09	3.3	3.35	0.94	0.89
266	Tongue clicking	A2M	0.53	4.08	3.55	3.45	0.98	0.88
267	Tongue clicking	A2M	0.67	3.97	3.45	3.41	0.95	0.91
268	Tongue clicking	A4M	0.8	4.42	3.83	3.79	0.94	0.92
269	Tongue clicking	A4M	0.67	4.56	3.49	3.43	0.9	0.87
270	Tongue clicking	A4M	0.53	4.33	3.44	3.3	0.96	0.84
271	Tongue clicking	A7F	0.67	4.44	4.06	4.01	0.96	0.95
272	Tongue clicking	A7F	0.73	4.42	4.28	4.27	1	0.99
273	Tongue clicking	A7F	0.73	4.58	4.4	4.38	0.99	0.98
274	Tongue clicking	A8F	0.53	4.87	4.77	4.73	1	1
275	Tongue clicking	A8F	0.8	4.91	4.71	4.7	0.99	0.99
276	Tongue clicking	A8F	0.67	4.85	4.77	4.74	1	0.98
277	Tongue clicking	A9F	0.8	4.36	4.05	4.02	0.98	0.95
278	Tongue clicking	A9F	0.87	4.43	4.16	4.15	0.99	0.95
279	Tongue clicking	A9F	0.87	4.4	3.99	3.96	0.96	0.95
280	Tongue clicking	A10F	0.6	3.71	2.33	2.24	0.88	0.78
281	Tongue clicking	A10F	0.4	3.56	1.8	1.77	0.81	0.71
282	Whistling	A1M	0.27	4.35	4.08	4.08	0.98	0.97
283	Whistling	A1M	0.87	4.41	4.33	4.32	1	0.98
284	Whistling	A1M	0.93	4.56	4.47	4.43	1	0.99
285	Whistling	A3M	0.93	4.75	4.5	4.43	0.99	0.96
286	Whistling	A3M	0.93	4.87	4.6	4.58	0.99	0.96
287	Whistling	A3M	0.87	4.81	4.5	4.48	0.99	0.98
288	Whistling	A4M	0.87	4.45	4.27	4.25	1	0.98
289	Whistling	A4M	0.8	4.59	4.54	4.51	1	1
290	Whistling	A4M	0.73	4.38	3.93	3.9	0.96	0.94
291	Whistling	A7F	0.93	4.44	4.16	4.07	0.99	0.94
292	Whistling	A7F	0.93	4.67	4.45	4.4	0.99	0.96
293	Whistling	A7F	0.8	4.45	4.09	4.08	0.98	0.95
294	Whistling	A8F	0.93	4.76	4.68	4.62	1	0.99
295	Whistling	A8F	0.6	4.81	4.67	4.64	0.99	0.98
296	Whistling	A8F	0.8	4.81	4.7	4.64	1	0.98
297	Whistling	A9F	0.93	4.62	4.56	4.55	1	1
298	Whistling	A9F	0.93	4.78	4.72	4.7	1	0.99
299	Whistling	A9F	0.87	4.76	4.6	4.6	0.99	0.98

## Appendix 2

**Table 6:** Table showing stimulus recognisability and quality scores for each individual stimulus in the Interoceptive States Point Light Displays (ISPLD) database. Recognisability Index (RI) was obtained in Validation Phase One (Free Labelling Task). Quality Index (QI), Selectivity Index (SI), Maximum Distractor Selectivity Index (SI+), Choice Rate (CR) and High-Quality Choice Rate (CR+) were obtained in Validation Phase Two (Label Selection and Rating Task). Actor ID includes the actor number (A1-A10) followed by their sex (M = male, F = female)

Stimulus	Stimulus Type	Actor ID	RI	QI	SI	SI+	CR	CR+
1	Breathlessness	A3F	0.37	2.86	0.64	0.51	0.67	0.5
2	Breathlessness	A3F	0.16	1.72	-0.63	-0.68	0.48	0.36
3	Breathlessness	A3F	0.16	2.98	1.37	1.34	0.74	0.57
4	Breathlessness	A3F	0.47	3.48	1.93	1.82	0.82	0.65
5	Breathlessness	A3F	0.16	2.88	1.39	1.33	0.7	0.58
6	Breathlessness	A3F	0.21	3	1.32	1.25	0.77	0.6
7	Breathlessness	A5M	0.42	2.63	0.73	0.68	0.69	0.53
8	Breathlessness	A5M	0.11	0.64	-2	-2.1	0.22	0.09
9	Breathlessness	A5M	0.26	2.82	0.55	0.52	0.73	0.48
10	Breathlessness	A6F	0.26	2.03	-0.03	-0.09	0.55	0.43
11	Breathlessness	A6F	0.42	3.55	1.29	1.19	0.81	0.56
12	Breathlessness	A6F	0.11	2.35	0.05	-0.06	0.6	0.42
13	Breathlessness	A7M	0.05	2.88	1.33	1.27	0.77	0.63
14	Breathlessness	A7M	0.16	1.81	-0.6	-0.7	0.54	0.33
15	Breathlessness	A8M	0.32	1.96	-0.69	-0.78	0.52	0.33
16	Breathlessness	A8M	0.37	2.49	-0.35	-0.48	0.61	0.33
17	Breathlessness	A9M	0.11	1.98	-0.56	-0.64	0.52	0.36
18	Breathlessness	A10F	0.16	2.91	1.24	1.22	0.73	0.63
19	Breathlessness	A10F	0.37	3.33	1.56	1.52	0.76	0.68
20	Cold	A1M	0.26	3.53	2.57	2.56	0.81	0.78
21	Cold	A1M	0.53	3.47	2.52	2.49	0.78	0.73
22	Cold	A3F	0.37	2.76	1.58	1.55	0.71	0.59
23	Cold	A3F	0.32	3.17	2.19	2.13	0.75	0.7
24	Cold	A3F	0.42	3.66	2.97	2.93	0.83	0.78
25	Cold	A5M	0.05	2.92	1.74	1.69	0.76	0.65
26	Cold	A5M	0.37	2.77	1.41	1.34	0.7	0.61
27	Cold	A5M	0.63	3.89	3.03	3.02	0.85	0.81
28	Cold	A5M	0.26	2.59	1.15	1.1	0.67	0.58
29	Cold	A6F	0.21	4.07	3.79	3.77	0.95	0.94
30	Cold	A7M	0.26	2.79	1.6	1.52	0.71	0.66
31	Cold	A7M	0.32	3.3	2.33	2.3	0.79	0.78
32	Cold	A7M	0.42	3.58	3	2.96	0.85	0.83
33	Cold	A8M	0.42	3.01	1.77	1.67	0.75	0.65
34	Cold	A8M	0	2.5	1.07	0.98	0.69	0.58
35	Cold	A8M	0.53	3.35	2.27	2.22	0.75	0.71
36	Cold	A9M	0.21	3.34	2.25	2.2	0.8	0.69
37	Cold	A9M	0.21	4.18	3.73	3.71	0.92	0.91
38	Cold	A10F	0.68	3.98	3.09	3.08	0.86	0.79
39	Cold	A10F	0.63	4.58	4.29	4.28	0.96	0.95
40	Cold	A10F	0.42	3.1	2.03	1.96	0.78	0.7
41	Fatigue	A5M	0.16	1.25	-1.19	-1.23	0.32	0.28
42	Fatigue	A6F	0.37	3.38	2.6	2.58	0.86	0.81
43	Fatigue	A3F	0.26	1.76	0.05	0	0.52	0.43
44	Fatigue	A4F	0.21	3.89	2.69	2.64	0.89	0.82
45	Fatigue	A4F	0.16	3.41	2.83	2.82	0.85	0.83
46	Fatigue	A4F	0.32	3.53	2.75	2.72	0.88	0.82
47	Fatigue	A5M	0.16	3.28	2.44	2.4	0.83	0.77
48	Fatigue	A6F	0	3.42	2.64	2.6	0.84	0.8
49	Fatigue	A6F	0.11	4	3.02	2.98	0.91	0.81
50	Fatigue	A7M	0.26	3.23	2.51	2.47	0.85	0.78
51	Fatigue	A7M	0.11	3.7	3.13	3.11	0.91	0.88
52	Fatigue	A10F	0.21	2.92	2.05	2.01	0.83	0.75
53	Fatigue	A10F	0.16	2.7	1.36	1.32	0.71	0.64
54	Hot	A2F	0.05	2.84	1.49	1.43	0.69	0.63
55	Hot	A2F	0.05	2.7	1.47	1.36	0.69	0.64
56	Hot	A3F	0.11	0.79	-1.45	-1.52	0.3	0.22
57	Hot	A3F	0	1.27	-1.1	-1.18	0.38	0.26
58	Hot	A3F	0	1.54	-0.29	-0.39	0.5	0.41
59	Hot	A3F	0	2.06	0.34	0.3	0.52	0.47
60	Hot	A5M	0	1.69	-0.24	-0.26	0.41	0.41
61	Hot	A5M	0.05	3.04	1.78	1.71	0.72	0.63
62	Hot	A5M	0.58	3.86	2.65	2.63	0.86	0.79
63	Hot	A6F	0.21	2.24	0.69	0.59	0.61	0.52
64	Hot	A6F	0.32	3	1.71	1.68	0.72	0.67
65	Hot	A6F	0.16	2.03	0.28	0.24	0.53	0.48
66	Hot	A6F	0.11	1.32	-1.14	-1.26	0.36	0.26
67	Hot	A7M	0.16	1.48	-0.54	-0.57	0.4	0.33
68	Hot	A1M	0.05	1.57	-0.36	-0.41	0.48	0.36
69	Hunger	A1M	0.21	2.21	0.09	0.01	0.61	0.42
70	Hunger	A1M	0.42	2.53	0.64	0.56	0.69	0.53
71	Hunger	A2F	0.37	2.65	0.91	0.88	0.71	0.55
72	Hunger	A2F	0.37	2.54	0.96	0.88	0.74	0.61
73	Hunger	A2F	0.11	2.08	0.3	0.27	0.63	0.46
74	Hunger	A3F	0.37	2.31	0.66	0.6	0.67	0.51
75	Hunger	A3F	0.26	2.54	0.8	0.77	0.7	0.54
76	Hunger	A4F	0.05	2.02	0.12	0.05	0.58	0.44
77	Hunger	A4F	0.05	1.2	-1.27	-1.33	0.38	0.25
78	Hunger	A4F	0.16	1.95	0.08	0.02	0.57	0.44
79	Hunger	A5M	0.05	1.59	-0.53	-0.57	0.47	0.36
80	Hunger	A5M	0.47	2.94	1.27	1.21	0.75	0.6
81	Hunger	A5M	0.16	2.5	0.85	0.79	0.69	0.59
82	Hunger	A6F	0.32	2.19	-0.12	-0.17	0.61	0.4
83	Hunger	A6F	0.47	2.57	0.69	0.56	0.71	0.49
84	Hunger	A6F	0.21	1.54	-0.84	-0.91	0.47	0.27
85	Hunger	A6F	0	0.3	-2.3	-2.46	0.13	0.03
86	Hunger	A7M	0.16	2.4	0.84	0.8	0.69	0.58
87	Hunger	A7M	0.26	2.25	0.52	0.47	0.66	0.47
88	Hunger	A8M	0.05	2.2	0.22	0.14	0.67	0.49
89	Hunger	A9M	0.05	2.03	-0.17	-0.27	0.58	0.35
90	Hunger	A9M	0.21	2.38	0.49	0.45	0.67	0.48
91	Hunger	A9M	0.26	2.36	0.53	0.46	0.65	0.5
92	Hunger	A10F	0.16	2.22	0.33	0.3	0.65	0.48
93	Itch	A1M	0.11	2.25	0.37	0.34	0.57	0.5
94	Itch	A1M	0.68	4.19	3.87	3.84	0.95	0.92
95	Itch	A1M	0.53	4.52	4.3	4.3	0.98	0.95
96	Itch	A1M	0.42	4.34	4.16	4.16	0.96	0.95
97	Itch	A3F	0.53	3.82	3.05	3.02	0.9	0.81
98	Itch	A3F	0.47	4.44	4.31	4.29	0.96	0.96
99	Itch	A3F	0.68	3.92	3.44	3.42	0.9	0.86
100	Itch	A5M	0.37	4.21	3.91	3.91	0.92	0.92
101	Itch	A5M	0.58	4.13	3.88	3.86	0.96	0.95
102	Itch	A5M	0.53	4.04	3.67	3.65	0.92	0.91
103	Itch	A6F	0.42	4.52	4.39	4.39	0.97	0.97
104	Itch	A6F	0.11	2.57	0.89	0.76	0.66	0.54
105	Itch	A6F	0.47	4.42	4.26	4.24	0.97	0.97
106	Itch	A7M	0.42	4.64	4.54	4.54	0.97	0.97
107	Itch	A7M	0.74	4.65	4.53	4.53	0.98	0.98
108	Itch	A8M	0.47	4.1	3.77	3.76	0.92	0.9
109	Itch	A10F	0.68	4.66	4.52	4.52	0.98	0.98
110	Itch	A10F	0.47	3.65	3.03	2.98	0.85	0.82
111	Nausea	A1M	0.11	1.95	-0.67	-0.83	0.51	0.31
112	Nausea	A1M	0.63	3.3	1.55	1.47	0.72	0.63
113	Nausea	A3F	0.42	2.53	0.48	0.38	0.64	0.5
114	Nausea	A3F	0.16	2.2	0.17	0.1	0.6	0.45
115	Nausea	A3F	0.11	1.95	-0.39	-0.49	0.52	0.36
116	Nausea	A5M	0.37	3.47	2.29	2.22	0.78	0.74
117	Nausea	A7M	0.47	3.71	2.3	2.29	0.85	0.73
118	Nausea	A8M	0.11	1.15	-1.8	-1.95	0.36	0.16
119	Nausea	A8M	0.32	1.71	-0.8	-0.91	0.46	0.29
120	Nausea	A8M	0	0.72	-2.14	-2.25	0.23	0.13
121	Nausea	A9M	0.42	3.52	2.39	2.33	0.81	0.74
122	Nausea	A9M	0.32	3.61	2.39	2.34	0.83	0.76
123	Satiety	A1M	0.11	1.6	-0.33	-0.38	0.48	0.39
124	Satiety	A1M	0.11	1.81	-0.18	-0.21	0.48	0.43
125	Satiety	A2F	0	2.45	1.09	1.05	0.64	0.58
126	Satiety	A2F	0	2.51	1.15	1.1	0.65	0.59
127	Satiety	A2F	0.05	1.65	-0.59	-0.68	0.41	0.34
128	Satiety	A3F	0.05	1.88	0.39	0.35	0.54	0.5
129	Satiety	A3F	0.05	1.84	0.28	0.23	0.5	0.45
130	Satiety	A3F	0	2.03	0.52	0.45	0.61	0.49
131	Satiety	A4F	0	1.01	-1.29	-1.4	0.3	0.2
132	Satiety	A4F	0	1.22	-1.09	-1.22	0.35	0.27
133	Satiety	A4F	0.11	1.72	-0.33	-0.44	0.47	0.34
134	Satiety	A5M	0.11	2.43	1.19	1.1	0.6	0.54
135	Satiety	A5M	0	2.03	0.31	0.23	0.55	0.44
136	Satiety	A5M	0	2.02	0.38	0.26	0.54	0.46
137	Satiety	A6F	0	2.39	0.95	0.92	0.57	0.55
138	Satiety	A6F	0	1.36	-0.65	-0.71	0.39	0.32
139	Satiety	A7M	0.05	1.53	-0.35	-0.43	0.47	0.39
140	Satiety	A7M	0.11	2.84	1.6	1.53	0.64	0.63
141	Satiety	A7M	0	1.61	-0.44	-0.51	0.45	0.41
142	Satiety	A8M	0	1.07	-1.39	-1.47	0.32	0.23
143	Satiety	A9M	0	1.34	-0.38	-0.42	0.43	0.36
144	Satiety	A9M	0.05	2.03	0.15	0.11	0.54	0.45
145	Satiety	A10F	0.16	1.39	-0.77	-0.9	0.38	0.26
146	Thirst	A1M	0	0.85	-1.69	-1.76	0.21	0.19
147	Thirst	A1M	0.05	1.02	-1.66	-1.79	0.35	0.2
148	Thirst	A1M	0	0.22	-2.79	-2.99	0.1	0.03
149	Thirst	A2F	0	1.95	0.18	0.05	0.6	0.46
150	Thirst	A2F	0	1.45	-0.76	-0.82	0.41	0.32
151	Thirst	A3F	0	0.72	-1.79	-1.83	0.25	0.16
152	Thirst	A3F	0	0.54	-1.69	-1.76	0.23	0.14
153	Thirst	A4F	0	1.14	-0.65	-0.71	0.4	0.34
154	Thirst	A5M	0	0.66	-1.83	-1.86	0.22	0.18
155	Thirst	A5M	0	0.74	-1.94	-2.07	0.25	0.15
156	Thirst	A5M	0	1.44	-0.71	-0.81	0.5	0.32
157	Thirst	A6F	0	0.69	-1.37	-1.41	0.22	0.21
158	Thirst	A6F	0	0.76	-1.88	-1.97	0.31	0.15
159	Thirst	A9M	0	0.27	-2.77	-2.91	0.1	0.06
