# Supplementary material for: Development and validation of the Interoceptive States Vocalisations (ISV) and Interoceptive States Point Light Displays (ISPLD) databases
Source: Behav Res Methods. 2025 Mar 31;57(5):133. doi: 10.3758/s13428-024-02514-0 (PMC11958399; doi:10.3758/s13428-024-02514-0)
Supplement: Supplementary file 1 — Supplementary file1 (DOCX 77 KB) [file 13428_2024_2514_MOESM1_ESM.docx]

**Supplementary materials 1**

**Welch tests for ISV stimuli**

As the assumption of homogeneity of variance was not met for the ANOVAs reported in the manuscript, one way Welch’s ANOVAs with Stimulus Type as the Independent Variable and the five stimulus quality scores as the Dependent Variables were also utilised. For all Dependent Variables, a significant effect of Stimulus Type was observed, consistent with the ANOVA analyses. Results of each test are reported in Table S1 below.

| Dependent variable | Degrees of freedom | Welch’s *F* | *p* |
| --- | --- | --- | --- |
| QI internal state | 6, 80.44 | 42.60 | < .001 |
| QI control state | 4, 50.39 | 12.14 | < .001 |
| SI internal state | 6, 80.42 | 161.47 | < .001 |
| SI control state | 4, 47.86 | 29.70 | < .001 |
| SI+ internal state | 6, 80.46 | 161.81 | < .001 |
| SI+ control state | 4, 47.70 | 29.99 | < .001 |
| CR internal state | 6, 79.68 | 169.88 | < .001 |
| CR control state | 4, 45.81 | 28. 97 | < .001 |
| CR+ internal state | 6, 79.72 | 214. 05 | < .001 |
| CR+ control state | 4, 45.63 | 27.10 | < .001 |

**Figure S1.** Significant interaction between Stimulus Type and Actor Sex for QI scores for Interoceptive State stimuli from the Interoceptive State Vocalisations stimulus set. Males and females did not differ significantly in QI for any stimulus type. The significant interaction appears to be driven by the pattern of QI scores across the stimulus types varying between males and females, such as breathlessness stimuli receiving the highest QI for males, but the third highest for females, and fatigue stimuli receiving the second highest QI for female actors, but the fifth highest for male actors.


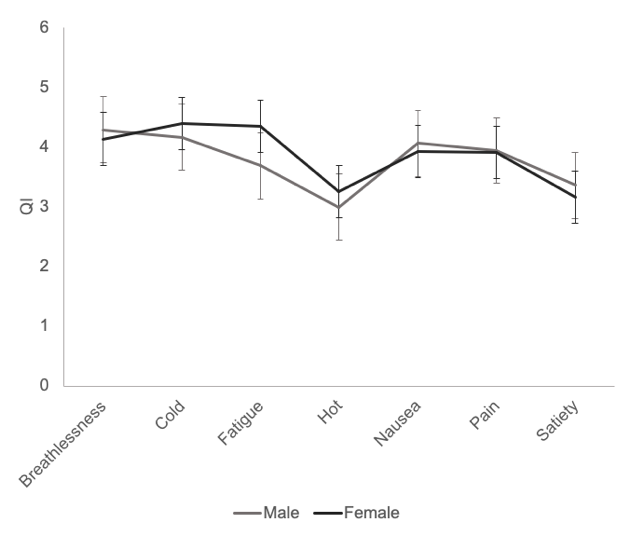


**Welch tests for ISPLD stimuli**

As the assumption of homogeneity of variance was not met for the ANOVAs reported in the manuscript, one way Welch’s ANOVAs with Stimulus Type as the Independent Variable and the five stimulus quality scores as the Dependent Variables were also utilised. For all Dependent Variables, a significant effect of Stimulus Type was observed, consistent with the ANOVA analyses. Results of each test are reported in Table S1 below.

| Dependent variable | Degrees of freedom | Welch’s *F* | *p* |
| --- | --- | --- | --- |
| QI | 8, 56.67 | 41.95 | < .001 |
| SI | 8, 56.55 | 38.56 | < .001 |
| SI+ | 8, 56.57 | 38.08 | < .001 |
| CR | 8, 56.26 | 35.88 | < .001 |
| CR+ | 8, 56.73 | 39.22 | < .001 |
